# Advances in sepsis diagnosis and management: a paradigm shift towards nanotechnology

**DOI:** 10.1186/s12929-020-00702-6

**Published:** 2021-01-08

**Authors:** Amit Pant, Irene Mackraj, Thirumala Govender

**Affiliations:** 1 Discipline of Pharmaceutical Sciences, College of Health Sciences, University of KwaZulu-Natal, Private Bag X54001, Durban, South Africa; 2 School of Laboratory Medicine and Medical Sciences, College of Health Sciences, University of KwaZulu-Natal, Private Bag X54001, Durban, South Africa

**Keywords:** Sepsis, Antimicrobial resistance, Nanotechnology, Nanodiagnostics, Nanotherapeutics

## Abstract

Sepsis, a dysregulated immune response due to life-threatening organ dysfunction, caused by drug-resistant pathogens, is a major global health threat contributing to high disease burden. Clinical outcomes in sepsis depend on timely diagnosis and appropriate early therapeutic intervention. There is a growing interest in the evaluation of nanotechnology-based solutions for sepsis management due to the inherent and unique properties of these nano-sized systems. This review presents recent advancements in nanotechnology-based solutions for sepsis diagnosis and management. Development of nanosensors based on electrochemical, immunological or magnetic principals provide highly sensitive, selective and rapid detection of sepsis biomarkers such as procalcitonin and C-reactive protein and are reviewed extensively. Nanoparticle-based drug delivery of antibiotics in sepsis models have shown promising results in combating drug resistance. Surface functionalization with antimicrobial peptides further enhances efficacy by targeting pathogens or specific microenvironments. Various strategies in nanoformulations have demonstrated the ability to deliver antibiotics and anti-inflammatory agents, simultaneously, have been reviewed. The critical role of nanoformulations of other adjuvant therapies including antioxidant, antitoxins and extracorporeal blood purification in sepsis management are also highlighted. Nanodiagnostics and nanotherapeutics in sepsis have enormous potential and provide new perspectives in sepsis management, supported by promising future biomedical applications included in the review.
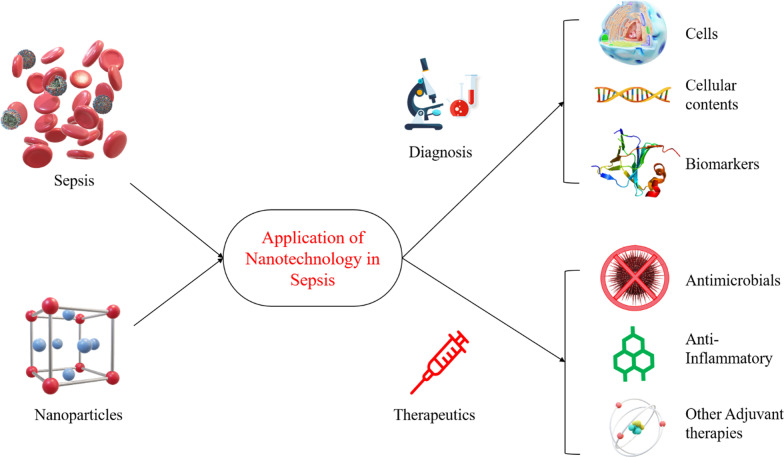

## Introduction

Sepsis is described as a syndrome consisting of complex pathophysiological and biochemical dysregulation, triggered by endogenous factors in response to the bacterial, viral, parasitic or fungal infections [1]. The definition of sepsis has recently been modified and updated, due to advancements in our understanding of the underlying pathophysiology based on molecular and clinical research. Based on the new definition, sepsis is currently defined as “life-threatening organ dysfunction, caused by a dysregulated host immune response to infection” [1, 2]. Currently, the World Health Assembly (WHA), the WHO’s decision-making body, recognizes sepsis as a significant threat to patient safety and global health and has intensified its approach to the prevention, diagnosis, and treatment of sepsis [3]. A recently published analysis, viz. ‘Global Burden of Diseases, Injuries, and Risk Factors Study (GBD 2017),’ has revealed that 48.9 million cases of sepsis and 11.0 million sepsis-related deaths, were recorded worldwide in 2017, representing approximately 20% of all global deaths [4]. In the USA, about 1.7 million sepsis cases and 270,000 sepsis-related deaths are reported each year [5]. Among children below five years of age, there is a significant burden of sepsis, causing 20 million cases and 2.9 million cases of sepsis globally every year [6]. Importantly, sepsis remains a major cause of intensive care unit (ICU) admissions in low and middle-income countries (LMICs); with mortality rates as high as 80% being reported from these regions [7]. Furthermore, emerging data from the current COVID-19 pandemic has shown a relationship between severe acute respiratory syndrome coronavirus-2 (SARS-CoV-2) and sepsis; characterised by organ damage attributable to viral invasion and systemic inflammatory responses [8–10]. Thus, sepsis remains a serious global health concern with life-threatening consequences, which requires urgent focus, especially in early diagnostics and innovative and effective therapeutic management.

Sepsis diagnosis in the early stages and timely therapeutic interventions are pivotal in improving clinical outcomes and reducing mortality [11, 12]. Conventionally, serum analysis and molecular techniques are used in the diagnosis of sepsis. The sepsis diagnosis is further complicated due to nonspecific signs and symptoms and can be challenging as there is no gold standard test that confirms the diagnosis [13]. The most common method for the detection of infectious pathogens in blood circulation is a blood culture test. Apart from this, several molecular techniques, including polymerase chain reaction (PCR), isothermal amplification methods, hybridization techniques and microarray, each with different sensitivity and specificity, are used for detection of infection-causing pathogens. Despite the urgent need for monitoring sepsis using biomarkers in clinical diagnosis, sepsis specific biomarkers are lacking to date. Although there are more than 170 biomarkers that have been reported for sepsis diagnosis, few are applicable in clinical diagnosis, and each has advantages and limitations [14–16]. Additionally, major clinical challenges are associated with heterogeneous patient population and variations in time of elevation of individual markers [17]. Conventional diagnostic approaches, mainly based on analysis of blood cultures, and specialized molecular diagnostic techniques such as polymerase chain reaction (PCR), isothermal amplification methods, hybridization and microarray techniques; are most often laboratory-specific, require trained personal, and are multi-step and resource intensive, with a restricted limit of detection (LOD) and specificity [11]. Thus, there is an urgent need for developing novel sepsis diagnostic approaches preferably adapted for accessibility for bedside diagnosis.

An early, validated diagnostic approach is vital in the design of personalized therapeutic management plans for better clinical outcomes. For instance, each hour delay in the initiation of antibiotic therapy increases the risk of mortality by 7–12% [18, 19]. Guidelines for sepsis management is focused on three major components (1) haemodynamic stabilization, (2) infection control, and (3) modulation of the septic responses [20]. Other interventions include nonspecific measures of organ support, such as oxygen therapy, mechanical ventilation, hemodynamic support, corticosteroids, and renal replacement therapy [21–24]. Sepsis management requires multimodal therapeutic approaches and is based on severity. A mild form with single organ system dysfunction can be managed by moderate support, while multiple organ dysfunction requires invasive therapies [23]. Although broad-spectrum antibiotics are integral in the management of sepsis, a major challenge associated with antibiotic therapy in sepsis is resistance by pathogens that adversely affects sepsis outcomes and increases mortality rates by approximately two-fold [7, 25]. Globally, an estimated 214,000 deaths due to neonatal sepsis are thought to be caused by resistant pathogens [26, 27].

Furthermore, apart from antimicrobial agents, novel adjunctive therapies such as synthetic antimicrobial peptides, anti-inflammatory agents, immunomodulators, blood purification and antioxidants are being explored and have shown some additive benefits on survival in sepsis patients [28]. Many of these alternative interventions for sepsis fails to reproduce efficacy in clinical practice. This disparity can be explained by sepsis-induced pharmacokinetic alteration, leading to poor biodistribution with compromised efficacy and safety of commonly used therapeutic agents, thus increased the risk of treatment failure or resistance development [29, 30]. Host response in sepsis triggers the systemic release of various cytokines, reactive oxygen species and other biomolecules which interfere with cellular functions hampering the pharmacokinetic profile of the therapeutic agents. Initial hypovolaemia resulting due to vasodilatation, capillary leak and the subsequent need for large volumes of infusion fluids lead to an increase in the volume of distribution (V_D_). Thus, endothelial dysfunction, increased in V_D_ and other associated conditions cause subtherapeutic plasma concentrations of antimicrobials and treatment failure [29]. Other challenges that impede efficacy include short half-life, lack of tissue or cell-specific targeting, and poor water solubility and bioavailability of many anti-inflammatories as well as antioxidant agents. Also, agents such as peptides exhibiting significant in vitro anti-inflammatory activity fail to reproduce the effect in vivo due to metabolism by cellular enzymes. Furthermore, the complex pathophysiology involving the cytokine storm with multiple pathways, needs a multipronged approach as a single drug may not be effective. Antitoxin drugs also encounter the challenges of the removal of bacterial toxins due to poor selectivity.

Nanotechnology presents significant benefits for overcoming the challenges mentioned above for the diagnosis and management of sepsis. Currently, nanotechnology-based solutions are being evaluated for the identification of infections, the identification of organ dysfunction and for providing solutions in point-of-care settings for diagnosis of immune dysregulation [31]. Nano-biotechnology based sensors, such as electrochemical, optical, magnetic and immunosensors, have been explored and emerging alternatives with enhanced sensitivity and specificity providing quicker and reliable results compared to conventional methods [11]. The physicochemical properties of nanoparticles (NPs) including size, shape and large surface-area-to-volume ratios favour longer circulatory half-lives and target specific biodistribution profiles, compared to the free drug, thus improving antimicrobial, anti-inflammatory and antioxidant activity. Importantly, NPs surfaces can be functionalized with multiple agents that target a specific type of cell. Many organic and inorganic based polymers and carrier components, used in nanosystems, have shown some antimicrobial and anti-inflammatory properties, which give added benefits in infection management. These added and synergistic antimicrobial activity helps to overcome antibiotic resistance and can be useful as an antibiotic sparing strategy [32]. Several researchers are using this phenomenon for developing hybrid nanoformulations, wherein therapeutic benefits can be maximized by targeting multiple physiological processes involved in sepsis progression. Such hybrid formulations have been synthesized by combining antimicrobial agents and anti-inflammatory activity which has survival benefits [33]. Hybrid nanoformulations can also provide the benefit of faster and sensitive diagnosis while photodynamic effects induced by nanosystems can be used simultaneously for extracorporeal blood disinfection [34]. Nanotechnology-based systems, therefore, provide a platform for innovation in areas such as target-specific drug delivery, reduction in drug-related adverse effects, and enhanced drug activity for effective diagnosis and management of sepsis.

Thus far, we found reviews on nanotechnology applications to sepsis regarding diagnostics [31] biosensors [11] and nanotherapeutics using promising applications of TLR inhibitors [35]. Furthermore, Khan et al. have summarized devices and surfaces fortified with metal and metal oxide nanoparticles, which is useful in controlling sepsis [36]. Thus far, only Yuk et al. have briefly summarized nanotechnological applications for both the diagnosis and management of sepsis [37]. However, several advancements in nanotechnology-based techniques have been recently reported for rapid sepsis diagnosis with improved sensitivity. Furthermore, considerable research in nanoparticle and nanoformulation based therapies have shown promising results in vitro and in vivo. Thus, there is a need for a systematic review of current updates on emerging nanotechnology-based diagnosis and management solutions for sepsis.

The focus of this review is to provide a comprehensive overview of nanodiagnostics, emerging and adjuvant nanotherapeutics, and nanotechnology-based preventive measures, evaluated for sepsis diagnosis and management. Initially, an updated consensus definition of sepsis, pathophysiological considerations, and challenges associated with sepsis diagnosis and management are presented. This is followed by a review of emerging advancements in nanotechnology for the development of diagnostic applications and nanoformulation of agents utilized for sepsis management. Future perspectives on the commercial potential of these products to improve the diagnosis and treatment of sepsis are finally highlighted.

## Sepsis

Defining sepsis is critical as it involves multiple pathological pathways during the progression of the disease. Thus, this section reviews the currently modified sepsis definition and pathophysiology of sepsis. For diagnosis and management, there is no one-size-fits-all approach and hence various applications are presented on current diagnostic and management practices, along with challenges thereof.

### Definition

The definition of sepsis has changed over the years since the terms systemic inflammatory response syndrome (SIRS), sepsis, severe sepsis, and septic shock were initially defined in 1991 by the American College of Chest Physicians and the Society of Critical Care Medicine [2]. Previously sepsis was defined as SIRS due to infection; “severe” sepsis was associated with organ dysfunction, hypoperfusion, or hypotension; and “septic shock” was linked to arterial hypotension despite adequate fluid resuscitation.

Currently, known as ‘Sepsis 3’—sepsis is defined as a ‘life-threatening organ dysfunction caused by a dysregulated host response to infection’ [2]. The current definition recognises severity and potential lethality which is initiated by an invading pathogen which results in a process in which the body’s defence response has a deleterious effect upon itself [38]. Organ dysfunction is represented by an increase in the SOFA score of 2 points or more, which is associated with in-hospital mortality higher than 10%. Importantly, septic shock, a subset of sepsis, is clinically identified by a ‘vasopressor requirement to maintain a mean arterial pressure of 65 mmHg or greater and serum lactate level greater than 2 mmol/l in the absence of hypovolemia’ [39]. Revised critical care guideline has been rolled out and is being implemented since the outbreak of COVID-19 [24].

**Fig. 1:**
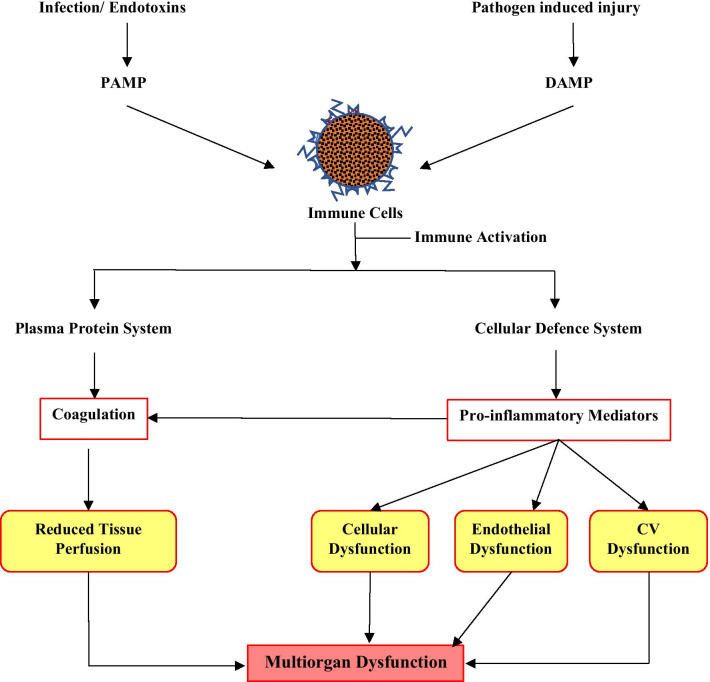
Pathophysiological changes in sepsis due to infection. Release of danger signal molecules (PAMP/ DAMP) activates the immune cells that mediate responses at the plasma protein level and the cellular level. Further downstream processes cause alterations in blood supply and oxygen consumption, leading to organ dysfunction

The current definition recognises the severity implicit by the term sepsis, and namely that sepsis is initiated by an invading pathogen and results in a process in which the body’s response is inappropriate. This pathophysiological response can culminate in multi-organ failure, usually due to a combination of cardiovascular, cellular, coagulation and endothelial dysfunction, aptly described as ‘the four horsemen of the septic apocalypse’ [38] (Fig. 1). It is the complexity of the multiple pathophysiological pathways of sepsis hinders the effective diagnosis and treatment of all the manifestations of sepsis. It has been recognised that timely diagnosis is essential so that treatment can be instigated as early as possible to ensure the best outcome, as a delay in treatment is associated with higher mortality [40, 41]. An understanding of the pathophysiology of sepsis is critical for both diagnostics and therapeutics, and an overview of key points are presented and revisited in more detail where applicable.

### Pathophysiology of sepsis

There is a general acceptance that the pathophysiology of sepsis is considered as an initial hyperinflammatory phase that lasts for several days, followed by a more protracted immunosuppressive phase [38, 42]. These two phases are associated with increased mortality and the current death distribution indicates two peaks; one of which manifests during the early phase albeit to a lesser extent, and another second peak after 2–3 months that continues to increase over the next 3 years [43, 44]. The peak mortality rate in the early period is attributed to an overwhelming inflammatory response, also known as a “cytokine storm,” which comprises fever, refractory shock, inadequate resuscitation, and cardiac or pulmonary failure. Mortality during the latter period is due to protracted immunosuppression with secondary infections, culminating in organ injury and/or failure. Despite sophisticated ICU care resulting in improved mortality, patients may still die at a later period or after several years owing to the persistent immunosuppression, immune dysfunction, or chronic catabolism [41].

It is therefore of interest to understand the pathophysiological events that lead to and underpin the cytokine storm and the primary role-players contributing to immunosuppressive state as it leads to increased mortality in patients. Hence the new definition correctly alludes to the immune response as the key focus area.

The early hyperinflammatory phase of the disorder is a series of steps leading to the cytokine storm. Early activation of both the innate and adaptive immune response is involved in the pathogenesis of sepsis [39]. The detection of the invading pathogen initiates the inflammatory response (IR). Host immune cells express pattern recognition receptors (PRP), on extracellular surfaces and in the cytosol, viz. Toll-like receptors (TLRs) and Nod-like receptors (NODs), respectively. TLRs are essential for pathogen detection, recognising ‘pathogen-associated molecular patterns’ (PAMPS) from pathogens, and recognising ‘damage-associated molecular patterns’ (DAMPS) from damaged endogenous cells. The overstimulation of TLRs by DAMPS may promote the IR in sepsis. NODs detect pathogens that invade the cytosol, leading to the formation of inflammasomes, promoting the production of inflammatory cytokines [40]. This promotes an ‘inflammatory state with activation of leucocytes, complement and coagulation pathways that underpin the endothelial, cellular and cardiovascular dysfunction that characterises sepsis’ [45]. Others indicate that disruptions in the normal homeostatic mechanisms of the immune and neuroendocrine systems during sepsis, alter cellular energy processes, disrupting endothelial and epithelial functions, which can ultimately cause dysfunction at the organ level [46] (Fig. 1).

The more protracted immunosuppressive phase is complex, multifactorial process stemming from immune cell depletion, due to uncontrolled apoptotic events as the primary mechanism of sepsis-induced immune suppression. Understandably if the key role players in the innate and natural IR is involved, the likelihood of the patient succumbing to secondary infections is greatly increased. Post-mortem findings are consistent with sepsis-induced immune cell apoptosis has now been confirmed in several post-mortem studies; it affects all age groups (neonatal, paediatric and adult populations) [42]. Indeed, Cao et al. (2019) describe the cells involved. Sepsis rapidly triggers profound apoptosis in cells representing nonspecific IR viz. macrophages/monocytes, dendritic cells, NK cells, γδ T cells, and those representing the specific IR viz. CD4 + T cells, and B cells. However, apoptosis of neutrophils is delayed, and Treg cells are more resistant to sepsis-induced apoptosis [38]. In particular, therapeutic perspectives targeting apoptosis through various strategies could improve survival in sepsis. Of importance is the consideration of what actually triggers this type of response instead of the patient overcoming the primary and secondary infections, which lead to death.

The complex pathophysiology of sepsis is a setback for the effective diagnosis and treatment of the disease; however, based on our current understanding of the pathophysiology of sepsis, novel diagnostic and therapeutic interventions are possible for a heterogeneous patient population. Biomarkers which include expression patterns may aid in stratifying patients into more homogeneous subgroups or in developing targeted therapeutic interventions. Furthermore, one must determine whether the patient is in the early hyperinflammatory phase of the disorder or has entered the more protracted immunosuppressive phase.

### Diagnosis of sepsis and challenges

Sepsis differs from the localized microbial infection in terms of host response which is dysregulated, generalized, with signs and symptoms, which are nonspecific [47]. For example, fever is a typical clinical reaction indicating the onset of a host response. At the same time, hyperthermia is common in critically ill patients, which is not always indicative of the presence of infection. Moreover, tachycardia and leukopenia are present in critically ill patients or may be due to other underlying pathological conditions. Most often, sepsis can sometimes be underdiagnosed in patients with hypoxia, and low platelet count but lacking evidence of infection or overdiagnosis can occur in postoperative patients on antibiotic therapy presenting with fever [13, 47]. Due to this, systemic inflammatory response syndrome (SIRS) criteria were proven to be unreliable and the application of such criteria is not recommended in sepsis diagnosis [20].

In addition to symptom-based diagnosis, culture reports from body fluids, preferably blood, is the most confirmatory and reliable means of diagnosis, but the assay time of 24 h to 48 h restricts their early clinical usefulness [48]. Unfortunately, around 30–40% of presumed sepsis cases of infected patients that are found to be culture-negative, which may be attributed to prior/ongoing antibiotic therapy or colony-forming units below detectable levels [20, 31]. Hence, due to the aforementioned diagnostic challenges, tracking using valid biomarkers which will allow for early therapeutic intervention, and improved patient outcomes is a key focus area in sepsis diagnosis. Biomarkers for sepsis are helpful in the identification of presence or absence of infection, the severity of disease condition, and the patient’s response to treatment [47]. C-reactive protein (CRP) is a widely studied acute-phase protein, triggered by both infection and inflammation leading to its augmentation, and despite high sensitivity, lack of specificity, restricts its application in sepsis diagnosis. Also extensively reported, procalcitonin (PCT) is perhaps a more specific marker than CRP, which is released during systemic inflammation caused by a bacterial infection [49]. However, other non-inflammatory diseases, such as burns, pancreatitis, or traumas, are found to influence the levels of both biomarkers.

Proven complexities and variable times of individual biomarker expression makes a single biomarker for sepsis diagnosis inappropriate, especially in critically ill patients. Development of combinations or panels of biomarkers for sepsis diagnosis is an emerging research area showing better reliability than individual biomarkers; however, further studies are needed to optimize the combinations of biomarkers. Also, patient stratification is critical, as we previously mentioned, a more personalised approach will overcome the attendant drawbacks of patient heterogeneity. Sepsis is associated with a cascade of pro-inflammatory and anti-inflammatory cytokines with variations in their occurrences according to the stages of sepsis. In the situation of a heterogeneous patient population, nanodiagnostic approaches have potential to serve as rapid and sensitive detection methods. Thus, patients can be stratified through early biomarker analysis that may help to implement the personalized medicines [50].

### Management of sepsis and challenges

Current Surviving Sepsis Guidelines provide up-to-date, evidence-based strategies in the management of sepsis, irrespective of etiologies [24, 51]. Unfortunately, it seems impossible to have a single multimodal and specific medicine as an ‘antisepsis’ [23]. Management of sepsis is multifaceted and includes early resuscitation, vasopressors, ventilation support, steroids, glucose control, anticoagulants and anti-inflammatories [24, 51]. Despite these guided approaches, there are many practical challenges in sepsis management, such as setting hemodynamic targets, selection of a type of fluid, as well as challenges in the application of the recommended therapies.

Fluid administration is a cornerstone in the management of hemodynamic instability, optimizing its administration is still challenging. Bolus administration of fluids may reduce arterial elastance leading to vasodilatation and the hyperdynamic state, while excessive fluid administration is associated with organ dysfunction and death [52]. Thus to avoid delayed hemodynamic stabilization, the early initiation of a vasopressor is recommended, but most of the vasopressors are lacking information on safe and effective doses [53]. Although antibiotic therapy is central in sepsis management, inappropriate therapy within the first 24 h leads to eight-fold higher, in-hospital mortality (Log-rank p = 0.0007). In contrast, a 74% higher progression in the inflammatory response, in inadequate empirical antibiotic therapy compared to adequate has been reported previously [54]. Other challenges associated with antibiotic therapy are hemodynamic alterations, leading to subtherapeutic dosing, and variations in resistance patterns amongst different regions [7, 52].

Despite the use of supportive therapy and timely administration, antibiotics are often ineffective and show little impact on lowering the mortality rate due to sepsis [55]. The immune paralysis associated with sepsis predisposes critically ill patients to secondary infections, including breakthrough infections, by multidrug-resistant (MDR) bacteria. Therefore, these patients require specific strategies directed to restore the function of immune response beyond the antibiotic therapy and standard supportive treatments. These adjuvant therapies can, therefore help the immune system by preventing immune-paralysis or attenuating inflammatory responses [23, 55]. Emerging data on adjuvant therapies ranging from extracorporeal blood purification techniques to various pharmacological approaches, including inhibition of proinflammatory cytokines, immunomodulation and antioxidant activity, presents novel sepsis research and in preclinical models found to be potentially active.

## Application of nanotechnology in diagnosis and management of sepsis

The application of nanotechnology-based solutions for clinical challenges is emerging and provides an array of opportunities in the diagnosis and management of critical illness [56]. Nanotechnology has shown potential in tackling microbial infection, including infections caused by resistance pathogens and thus revolutionizing the antimicrobial field [32]. Herein we discuss all these nanotechnology-based solutions in sepsis diagnosis and management by categorizing them as per their application and mechanism.

### Nanotechnologies in diagnosis

Diagnosis of sepsis is often based on biosensors that measure biological or chemical reactions. The biosensors are devices that generate a signal in proportion to concentrations of analyte in biological samples. Biosensors, in general, are composed of different components such as analyte, bioreceptors, signal transducers and display panels [57]. Biosensors have the ability to measure minuscule signals from various bodily fluids using a small number of samples [58]. Nanotechnology-based biosensors provide a novel approach to diagnostics with improved sensitivity for biomarkers and processing time without the requirement of specialized skills. The biomarkers in sepsis management are often evaluated for diagnostic, prognostic, monitoring, surrogate and stratification purposes [59]. A small set of biomarkers have been successful in clinical diagnosis for sepsis, which includes CRP, PCT, and Interleukin-6 (IL-6) [15, 60]. We describe the application of nanotechnology with regards to the detection of the biomarkers mentioned above.

Advancement and use of nanomaterials to detect various biomarkers have provided a new paradigm for the development of novel biosensors with the capability to monitor the level of any biomarker in different biological media such as whole blood, plasma, serum, cellular fluid or any other. A variety of analytical devices employed for sepsis diagnosis comprises different types of biosensors such as electrochemical, immunosensors and others. Herein, we discussed applications of nanotechnology in the development of biosensors mentioned above in detail with their features.

#### Electrochemical sensors

**Fig. 2:**
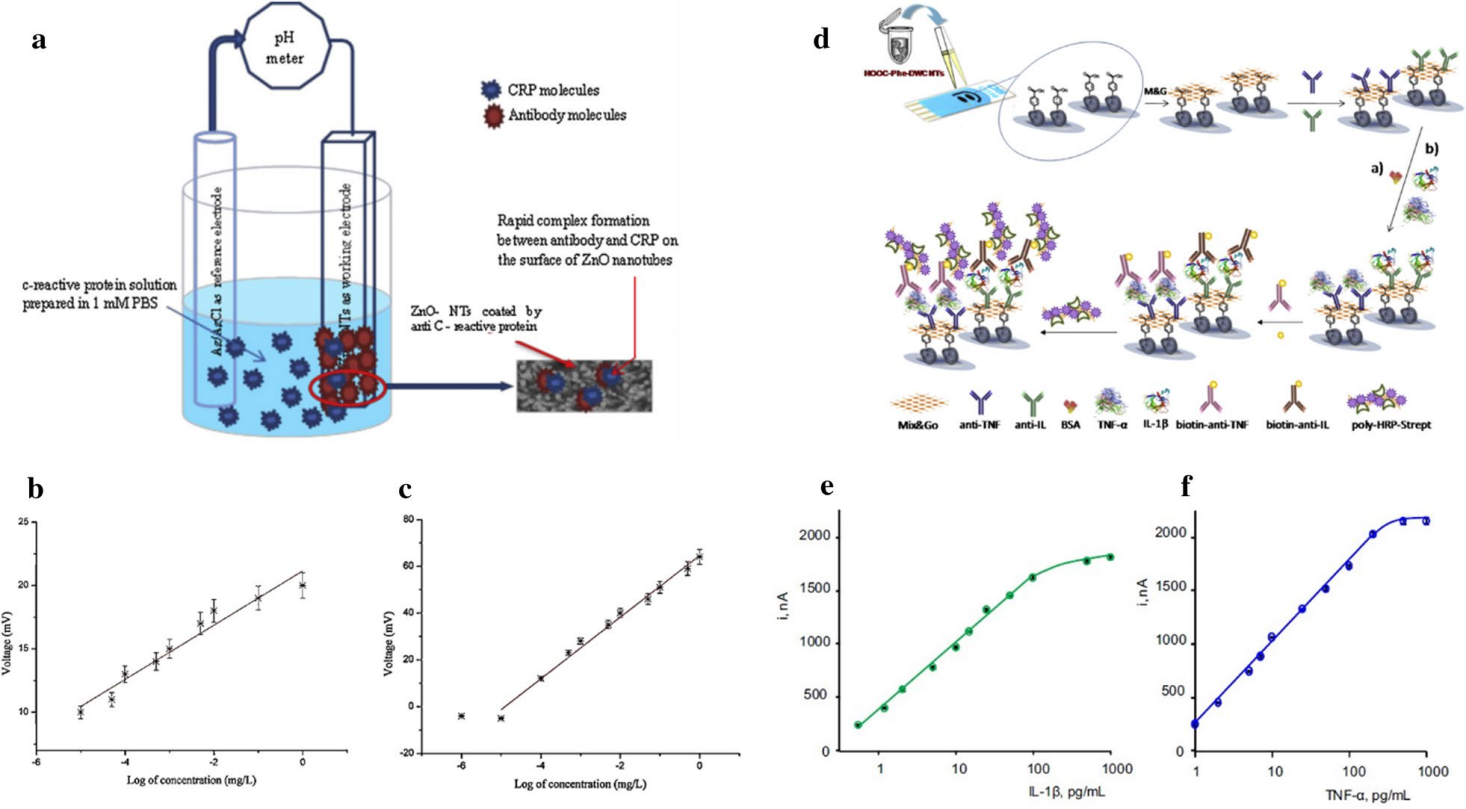
Schematic presentation of general protocol and principle of different electrochemical sensors. **a** Potentiometric antibody immobilized ZnO nanotubes-based biosensor for the detection of CRP; the calibration curve of bare ZnO nanotubes for CRP-antigen (**b**) and antibody immobilized ZnO nanotubes for CRP (**c**) [64]. **d** Different steps involved in the preparation of the dual electrochemical immunosensor for multiplexed determination of IL-1b and TNF-α cytokines. Calibration plots for IL-1b (**e**) and TNF-α (**f**) obtained with the dual poly-HRP-Strept-Biotin-anti-IL-IL1b-anti-IL-Phe-DWCNTs/SPCE and poly-HRP-Strept-Biotin-anti-TNF-TNF-α-anti-TNF-Phe-DWCNTs/SPCE immunosensor [68]

The electrochemical detection method has been widely used in portable biosensor devices and comprise of a chemical (molecular) recognition system and a physicochemical transducer in which the electrochemical sensor transforms chemical responses into an analytical signal [61]. Electrochemical biosensors applications are more commonly used for the detection of various biomarkers due to their sensitivity, selectivity, and reproducibility (Cho et al., 2018). CRP is a common sepsis biomarker, which is released either in response to infection or cytokine stimulation, in particular IL-6, during inflammation. The exact role of CRP in inflammation is unknown, but it may bind with bacterial components, enabling clearance by macrophages [15]. The level of CRP in a healthy individual is less than 10 mg/L, but the initial rise is reported in 4–6 h after tissue injury and peaks by several hundred folds within 24–48 h [62]. CRP exhibits good correlation with the severity of the infection and thus helps in early diagnosis in sepsis patients [63]. Ibupoto et al. (2012), for the first time, fabricated ZnO nanotubes functionalized with monoclonal anti-C-reactive protein clone CRP-8 (antibody), by a simple physical adsorption method used for the detection of CRP (Fig. 1). ZnO exhibits distinctive properties such as high isoelectric point (IEP) of 9.5 enabling the low IEP biomolecules (CRP) to bind with the ZnO surface, higher resistance for dissolution at the normal biological pH of 7.4, as well as higher polar properties contributing for about 60% ionic bonding characteristics. Application of ZnO nanomaterial in electrochemical biosensors are facilitated by high IEP, potentiating strong bonding with the immobilized antibody, and the piezoelectric property helps to generate the voltage along the CRP induced charged environment (Fig. 2a–c). Additionally, small size, extended surface to volume ratios, simple enzyme immobilization techniques and fast flow of the analyte through the sensors proved to be more rapid, sensitive, selective and reproducible relative to those of bulk ZnO devices. Antibody immobilized ZnO nanotubes based sensors, have shown a linear range of detection from 1.0 × 10^−5^ to 1.0 × 10^0^ mg/L [64]. Additionally, Gupta et al. (2014) reported on highly sensitive, selective, facile, low cost and label-free electrochemical sensing, using a carbon nanofiber-based biosensor platform. Functionalized carbon nanofiber tip surface and probe (anti-CRP) immobilization were developed for the detection of CRP and was shown to detect CRP within a clinically relevant range of ~ 90 pM or ~ 11 ng/ml [65].

PCT has emerged as a sepsis biomarker that differentiates bacterial infections from inflammatory responses, contrary to CRP, which lacks specificity for bacterial infections. PCT is a prohormone (peptide precursor) of calcitonin, secreted by the thyroid in a healthy person, and the presence of bacterial toxins can significantly elevate the serum PCT levels. To estimate the PCT levels, Liu and Wang (2015) developed an electrochemical immunoassay based on gold nanoparticles and ferrocene. They created a sandwich structure by using ferrocene-modified-Au-nanoparticles, labelled with PCT antibody (AB-I) as an electrochemical sign probe, and PCT-antibody (Ab-II)-modified gold electrode as an immunosensor, both reacting with PCT [66]. The properties of gold nanoparticles include a sizeable specific surface area, biocompatibility, low electrical resistance and the ability to load more ferrocene to help to enhance the electrochemical amplification signal, which accelerates electron transfer between the probe and the electrode. The PCT immunosensor exhibited excellent linearity and the limit of detection was 0.8 pg/mL in clinical samples and did not vary when compared to detection using ELISA, thus showing reliability. Furthermore, Liu et al. (2019) reported on an ultrasensitive electrochemical immunosensor, based on gold nanoparticles enhanced tyramide signal amplification (AuNPs-TSA) for the detection of PCT. The immunosensor is composed of a nanocomposite prepared from gold nanoparticles, horseradish peroxidase (HRP), polyethylene glycol and detection antibody (AuNPs-HRP-PEG-Ab2) and tyramide signal amplification (TSA). The detection range of this electrochemical immunosensor is reported to be between 0.05 and 100 ng/mL and with an ultralow limit of detection (LOD) of 0.1 pg/mL. This novel immunosensor offers benefits for PCT detection in differentiating bacterial and nonbacterial infections along with reproducibility, low LOD and notable sensitivity [67]. This immunosensor has the potential for clinical application and has been analysed for the detection of PCT in human serum samples consuming lower volumes. Similarly, Sánchez-Tirado et al. (2017) used a sandwich-type immunoassay in the development of a sensitive electrochemical dual-platform, with improved analytical performance, for the simultaneous determination of IL-β1 and TNF-α. Sandwich immunoassays, with amperometric signal amplification by using poly-HRP-streptavidin conjugates and hydrogen peroxide as HRP substrate and hydroquinone as a redox mediator, has been used for detection of both cytokines in serum or saliva. The general protocol for the development of electrochemical immunosensor is given in Fig. 2d and calibration plots are in Fig. 2e and f. The significant advantage of the dual immunosensor is an approximate four-fold reduction in assay time and 40-fold reduction in reagents consumption compared to ELISA protocol [68].

A new group of nanomaterials, the nanozymes, are emerging as alternatives to natural enzymes by overcoming the challenges of natural enzymes, such as a conserved working environment, stability, storage and cost. Recently Mahmudunnabi et al. (2020) summarized the applications of nanozymes in electrochemical biosensors for the detection of disease biomarkers along with bacterial species [69]. Such nanoenzyme based strategy has been reported to detect CRP in blood by citicoline-bovine serum albumin conjugates and aptamer-functionalized gold nanoparticles nanozymes with high accuracy and sensitivity [70]. Moreover, the use of nanozymes-based electrochemical biosensors have the potential to revolutionize on-site detection of a bacterial pathogen [71].

#### Immunosensors

Immunosensors are analytical devices that use specific antibody (monoclonal, polyclonal and recombinant antibodies) antigen reactions and provide a sensitive and selective tool for the determination of immunoreagents, which can be applied to sepsis biomarkers successfully. Application of immunosensors in disease diagnosis is an attractive option due to its high affinity to antibodies, low dissociation constant, signal amplification, high sensitivity, simplicity in fabrication, low cost, reproducibility and reliability. Conventional immunosensors using classical antibodies to detect clinically relevant antigens face challenges like sensitivity and operational conditions. Emerging nanotechnology is promising in detections of biomarkers using immunological assays with an enhanced intensity of the electrochemical signal. Nanobodies (Nbs), termed single-domain antigen-binding fragments, offer distinct advantages of smaller size, solubility, stability, strict monomeric behaviour and antigen specificity, beneficial for developing biosensors, and understanding biological processes, as well as for the generation of innovative therapeutics for the treatment of diseases.

**Fig. 3:**
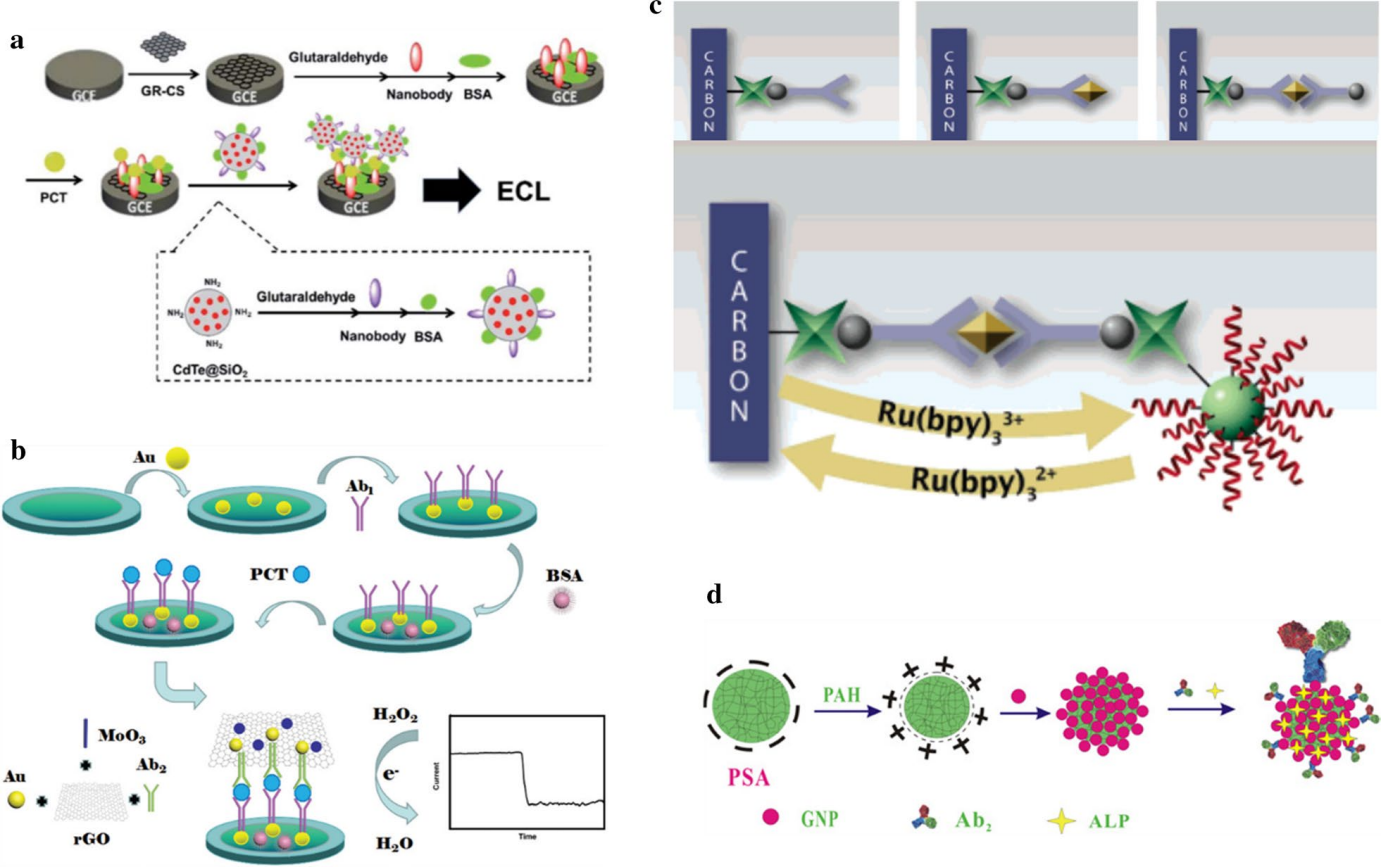
Schematic representation of the sandwich immunoassays: **a** NbI-GR-GS-GCE as a PCT capturing sensor and CdTe@SiO2/NbII to detect the PCT by ECL. The preparation of the CdTe@SiO2/NbII is also shown (dashed line box) [72]. **b** Fabrication of the electrochemical immunosensor for the detection of PCT [73] **c** Principle for Electrochemical Immunoassay Based on Poly[G]-Functionalized Silica NPs [74] **d** Assembly procedure of ALP-Ab2-GNPs/PSA bioconjugates [75]

Li et al. (2014) explored sensitive and specific PCT immunosensing, by combining characteristic Nbs, identified and isolated from a camel, with the advantageous silica-coated CdTe QD nanoparticles (CdTe@SiO_2_) for the nanoparticle-assisted signal amplification to generate a highly sensitive detection method with significantly increased signal amplification (Fig. 3a). The use of this sandwich immunoassay with NbI as a capture antibody for the immobilization on chitosan–graphene nanocomposite (GR–CS) modified glassy carbon electrode (GCE) as a PCT capturing reagent (NbI-GR-GS-GCE) and NbII as a detection antibody for conjugation with CdTe@SiO_2_ nanoparticles for PCT detection (CdTe@SiO_2_/NbII) yields more signal intensity of about fourfold. A detection limit for PCT was low as 3.4 pg/mL with a linear relationship with the concentration of PCT and was successfully tested in clinical samples as well [72]. Recently, Liu et al. (2019) reported another type of sandwich-type electrochemical immunosensor for the detection of PCT constructed by layer-by-layer modification of the glassy carbon electrode with new label nanocomposites. A new nanocomposite hybrid was designed by combination of MoO_3_/ Au@rGO and a schematic representation is given in the figure below (Fig. 3b). These transition metal oxides have been used as co-catalysts due to a synergistic effect or bifunctional mechanism. MoO_3_ nanorods provide improved electrocatalytic activity and stability for the oxidation–reduction reaction, while the Au/graphene nanosheet as the signal amplification material, exhibits excellent sensitivity and detection limits due to their enhanced electron transfer and catalytic activities. The electrochemical immunosensor exhibits a wide working range from 0.01 pg/mL to 10 ng/mL and a detection limit of 0.002 pg/mL. Thus, both immunosensors mentioned above, with highly sensitive and specific PCT detection, have an application for point-of-care diagnostics of sepsis and systemic inflammation processes [73].

Apart from PCT, tumor necrosis factor-α (TNF-α), a potent cytokine biomarker, is also involved in a wide range of pathological and physiological processes, acting as an endogenous mediator for inflammation and immunity. Highly sensitive methods of detection for TNF-α is of prime importance as its occurrence in biological samples is very low. Therefore, Wang et al. (2006) developed a sensitive biosensor involving a dual-signal amplification by poly[G]-functionalized silica NPs and catalytic oxidation of guanine (G) for the detection of TNF-α. The poly[G]- and avidin-functionalized silica NP label was prepared by covalent binding of poly[G] and avidin to the silica NP surface using the conventional coupling reagent (Fig. 3c). The dual signal amplification for the detection of TNF-α is accomplished via an immunological reaction. In an initial step, sandwich immunoreaction between poly(guanine)-functionalized silica NP label accumulates a large number of guanine residues on the electrode surface followed by the introduction of Ru(bpy)_3_^2+^ that leads to oxidation of guanine and thus improves anodic current [74]. This dual-signal amplification by poly[G]-functionalized silica NPs and catalytic oxidation of guanine can detect TNF-α at a lower concentration of ~ 0.05 ng/ mL.

Another method of detection of TNF-α, using electrochemical immunosensor based on alkaline phosphatase functionalized nanospheres, was reported by Yin et al. (2011). A multi-enzyme functionalized label was prepared by assembling gold nanoparticles on the surface of poly (styrene-acrylic acid) (GNP-PSA) nanospheres that conjugate alkaline phosphatase (ALP) (Fig. 3d). TNF-α antibody was immobilized on the composite prepared by electro-polymerization of polyaniline doped with poly (acrylic acid) at the glass carbon electrode [75]. The amperometrically measured electrochemical signals were linearly related to the antigen concentration (0.02–200.00 ng/mL) with the LOD of 0.01 ng/mL. A novel immunosensor based on alkaline phosphatase is more sensitive, stable and reproducible for detection of TNF-α at a lower concentration and may have future applications in diagnosis.

Molinero-Fernández et al. (2020) recently presented CRP detection by micromotor-based immunoassay (MIm) for early sepsis diagnosis in plasma of preterm infants with suspected sepsis. Micromotors constructed by three layers, namely, an outer layer for antibody functionalization, an intermediate Ni layer for magnetic guidance and stopped-flow operations and an inner catalytic layer of PtNPs for catalytic bubble propulsion [76]. The outer layer is biofunctionalized with carbon black (CB), reduced graphene oxide (rGO) and multi-walled carbon nanotubes (MWCNTs). The most attractive features of this newly reported immunoassay are the rapid, reliable detection using low sample volume (< 10 μL) and without dilution. However, validation of this immunoassay for the detection of CRP levels in the adults is awaited.

#### Miscellaneous nanosensors

More profound insights and knowledge of pathophysiological changes involved in sepsis, open up new opportunities in early diagnosis of sepsis. Recent advancements in diagnostic tools and emerging techniques, in a combination of different nanoparticles, provide rapid, highly selective and sensitive detection methods. Apart from the aforementioned electrochemical biosensors and immunosensors, other diagnostic approaches which utilize the principals of optical and magnetic resonance properties in conjunction with nanoparticles, have been reported, with a wide range of detection from protein biomarkers to pathogens. In an in-depth review, Mocan et al. (2017), reported on the different optical nanosensors, efficiently detecting pathogenic bacteria by various methods [77]. There is a series of articles on the application of T2 magnetic resonance-based techniques, combined with nanoparticles, for the detection of fungal species (*candida*) in clinical samples, which were also evaluated in clinical studies [78–80]. Hu et al. (2016) reported on CRP detection using immunofluorescent nanospheres (containing 332 ± 8 CdSe/ZnS quantum dots) that are stable for a more extended period (6 months) with an optical detection method [81]; while Kitayama and Takeuchi (2014) reported on CRP detection by grafting poly (2-methacryloyloxyethyl phosphorylcholine) onto gold nanoparticles [82]. Additionally, superparamagnetic iron oxide nanoparticles (SPIONs)-based biosensors platform have also been developed for the detection of changes in cellular uptake processes, using a contrast agent which is used in MRI [83].

In the recently reported study, ROS detection was performed by a dual-mode biosensor using both magnetic relaxation switching (MRSw) and fluorescence-based detection methods [84]. For MRSw based detection, initially, PEGylated bilirubin (PEG-BR)-coated SPIONs (PEG-BR@SPIONs) were developed by simple sonication via ligand exchange which was later coated with a near infra-red (NIR) fluorescent dye. Thus, the report revealed the possibilities of detection of ROS by a combination of different methods. This dual detection approach may have further potential for future application in diseases with excessive ROS production.

The simultaneous improvement in the exploration for novel biomarkers using nanotechnology with different diagnostic and prognostic potential is key in strengthening sepsis diagnosis. There are few novel biomarkers which have been tested for acute infections which are promising. These include citrullinated histone H3 (CitH3), soluble triggering receptor expressed on myeloid cells-1 (sTREM-1), soluble urokinase-type plasminogen receptor (suPAR), proadrenomedullin (pro-ADM), and presepsin [15, 85]. Considering the scope and limitation of the article, we will highlight the recent most promising novel biomarkers.

The existence of excessive neutrophil extracellular traps (NETs) has been explored in sepsis. NETs are of physiological significance with the ability to immobilize and kill a broad range of pathogens. However, NETs are also responsible for organ damage through interferance with tissue function, thrombosis and the autoimmune system. The NETs are often associated with citrullinated histones (CitH3) in the extracellular space of neutrophils [85]. Although the pathophysiological mechanism of CitH3 in sepsis is unknown, it has been shown to contribute to endothelial dysfunction, which is exarcerbated through positive feedback. Importantly, CitH3 concentration in blood can be detected as early as 30 min after endotoxic shock, and remains detectable for around 24 h, making it a more reliable biomarker compared to PCT and interleukin-1β and interleukin-6 [86]. The applicability of detecting CitH3 with high sensitivity has been recently demonstrated by Park et al. (2019) by constructing the “integrated nano optoelectronic biosensor” (iNOBS) device. The iNOBS device utilizes the combined effects of nanoscale plasmonic and photoelectronic effects for detection of CitH3. The iNOBS device was constructed using gold nanohemispheres (AuNHs), functionalizing with high-affinity CitH3 monoclonal antibodies incorporated with a photoconductive channel of localized surface plasmon resonance (LSPR) sensors layer above molybdenum disulfide (MoS2). This newly constructed iNOBS-based label-free binding assay demonstrated highly sensitive detection of CitH3. Key features of this device include 250-fold lower LOD (0.87 pg mL^ −1^ (56 fM)) than ELISA, an extended dynamic range [105], least processing time (20 min), and a requirement of small sample volume (2.5 μL). The detection of CitH3 was also performed with samples from the CLP-induced sepsis mouse model and showed a 100-fold faster measurement than the conventional western blot technique [87]. This study finding and emerging reports on the reliability of CitH3 demonstrate potential for its application for point-of-care clinical settings. A brief overview of the general assembly and limit of detection for the above-mentioned sensors are summarized in Table 1.

**Table 1:** Summary of different miscellaneous nanosensors using optical and magnetic detection method for various targets

Type of detection	Target	Components of sensor	Limit of detection	References
Optical detection	C-reaction protein	Fluorescent nanosphere (FN) contains 332 ± 8 CdSe/ZnS quantum dots (QDs) conjugated with antibodies to produce immunofluorescent nanosphere (IFN)-based lateral flow test strip	27.8 pM	[81]
Optical detection	C-reaction protein	Poly(2-methacryloyloxyethyl phosphorylcholine)-grafted AuNPs (PMPC-g-AuNPs)	∼50 ng/mL	[82]
Optical detection using novel quantitative susceptibility mapping (QSM) MRI	Uptake of superparamagnetic iron-oxide NPs by macrophages	–	–	[83]
T2 magnetic resonance	*Candida* spp.	Iron oxide nanocrystals embedded in a polymer matrix were conjugated to aminated DNA oligonucleotides	1–3 CFU/ml	[79]
Magnetic relaxation switching	Reactive oxygen species (ROS)	Infra-red (NIR) fluorescent dye loaded onto PEGylated bilirubin (PEG-BR)-coated SPIONs (PEG-BR@SPIONs)	31.49 μM	[84]
Combined plasmonic and photoelectronic detection	Citrullinated histones (CitH3)	Gold nanohemispheres (AuNHs), functionalizing with CitH3 antibodies incorporated with photoconductive channel above molybdenum disulfide (MoS_2_)	0.87 pg/mL	[86]

### Nanotechnology in antimicrobial treatment

The twenty-first century is becoming the “post-antibiotic” era due to the evolution of drug-resistant pathogens, as well as a dry pipeline in antibiotic research. The search for new and safer antibacterial drugs is at risk due to low returns; thus, the focus has shifted to targeted drug delivery to enhance local potency, while simultaneously reducing the untoward effects of antimicrobials. Application of nanotechnology in antimicrobial drugs provides distinct benefits other than the structural features, such as overcoming resistance [88] while avoiding its further development and causing fewer side effects than conventional antibiotics. Furthermore, nanoparticle formulations can prolong the half-life of the loaded antibiotic and serve as a sustained-release system, reducing the frequency of administration and improving therapeutic index [89]. Also, active targeting of an antibiotic to bacteria in an infected tissue is another strategy that can enhance the therapeutic index of antibiotics, while as vaccine adjuvant or delivery vehicles were shown to evoke more efficient immune responses. Besides this, many nanomaterials possess potent inherent antimicrobial properties than their bulk form, combating antibiotic resistance. Of significance, the potential of some antimicrobial nanotherapeutics is advantageous in inhibiting biofilms generation and targeting intracellular pathogens [32]. This section describes the nanotechnology applications broadly in different categories such as with antibiotics, antimicrobial peptides and other antimicrobials.

#### Antibiotic loaded nanoformulations

**Fig. 4:**
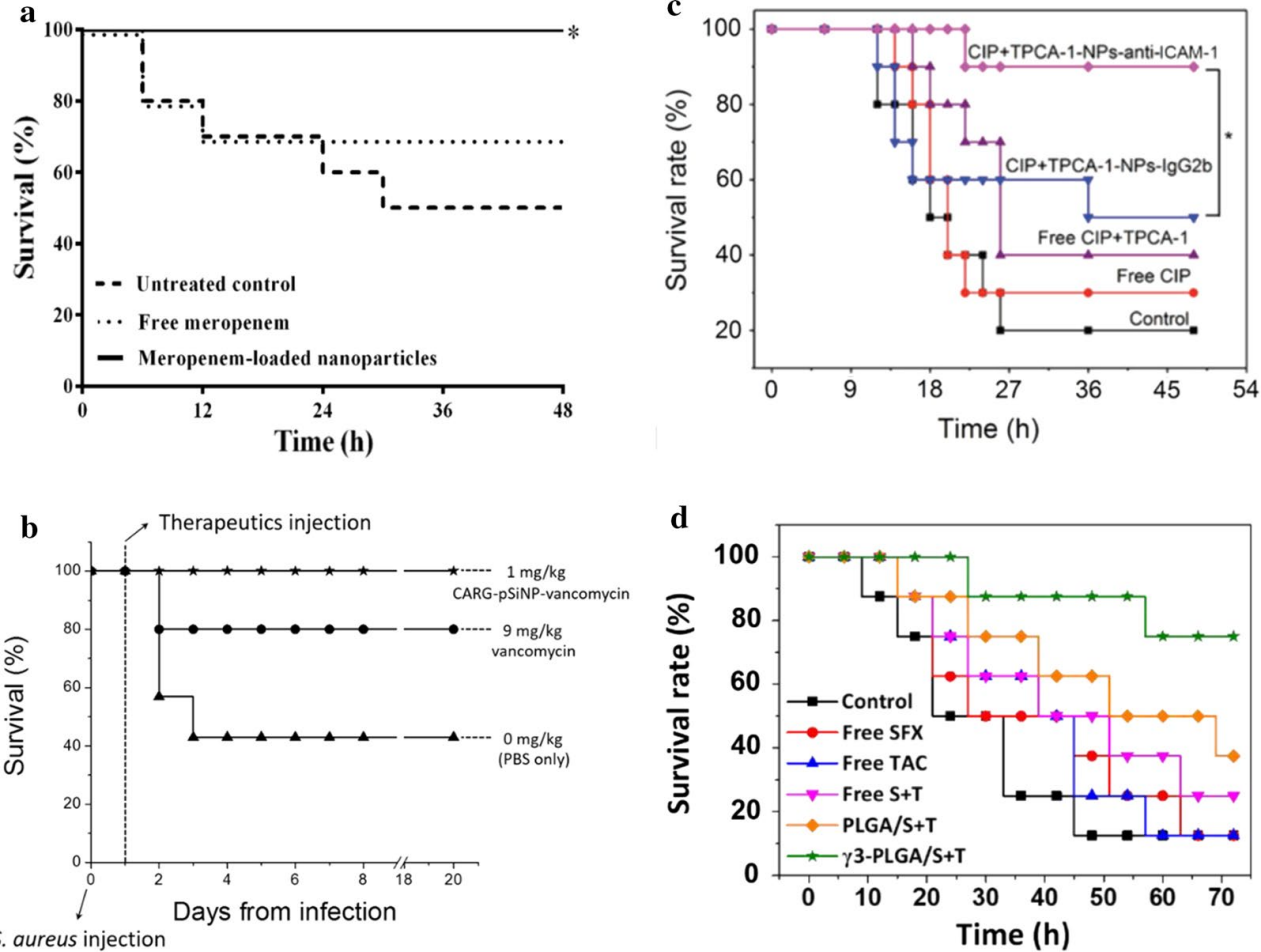
Effect on survival by different nano-antibiotic formulations. **a** Meropenem-loaded nanoparticles [91] **b** CARG-pSiNP-vancomycin [89] **c** CIP + TPCA-1-NPs-anti-ICAM-1 [94] **d** γ3-PLGA/S + T NPs [95]

Surviving sepsis guidelines strongly recommend rapidity in the administration of broad-spectrum antibiotics such as carbapenems or extended-spectrum β-lactamase inhibitors combinations [51]. Despite its broad-spectrum antibacterial activity with favourable safety, carbapenems possess certain limitations in terms of emerging resistance, short circulation half-life and administration of high dosage. A strategy to counter carbapenem resistance and improve therapeutic efficacy, carbapenems-loaded gold nanoparticles were reported to show a several-fold reduction in carbapenem MICs (minimum inhibitory concentration) [90]. Similar findings have been reported by Abdelkader et al., wherein, encapsulation of a time-dependent antibiotic, meropenem, into chitosan-based nanostructures using an ionic gelation method, demonstrated antibacterial potency against resistant pathogens, as well as improved pharmacokinetics in a septic animal model. This improved antibacterial potency can be attributed to the polycationic nature of chitosan (CS), which enhances interactions with the negatively charged bacterial cell walls and cytoplasmic membranes, thus facilitating higher drug penetration into the bacterial cells. The incorporation of meropenem into CS nanoparticles shows a statistically significant reduction in bacterial count in blood (CFU/mL), and survival rate (100%) with the drug-loaded nanoparticle dispersions during the 48 h, which was significantly higher than the untreated control group (*p* < 0.05) [91] (Fig. 4a).

However, for antibiotics such as Vancomycin, having a partially concentration-dependent activity [51]; appropriate pharmacodynamic targets, improved tissue penetration and optimal clinical outcomes, are dependent on the trough concentration. The alternate strategy to increase drug potency locally is to implement infection site-specific delivery of antibiotics, which may also reduce antibiotic-related systemic adverse events. This strategy has been studied by Hussain et al. (2018) who show the therapeutic delivery of vancomycin-carrying porous silicon nanoparticles (pSiNPs), bearing cyclic 9-amino acid peptide CARGGLKSC (CARG) targeting *S. aureus*-infected tissue, in a mouse lung infection model [89]. *S. aureus* though considered as an extracellular pathogen; available data supports its invasion of intracellular compartments, where it remains protected from host immune response, as well as antibiotics, leading to persistent infections or frequent relapse. CARG thus offers benefits of targeting intracellular pathogens along with biocompatibility, safety and high loading capacity. However, comparing the delivery of pSiNPs formulations with CARG, the delivery of CARG-pSiNPs was > fourfold effective in infected tissues than pSiNPs formulation. A vancomycin delivery through CARG medicated nanosystem was ~ tenfold effective than the free drug. The survival rates were 100% for CARG-pSiNP-vancomycin (1 mg/kg; n = 5), while a higher dose of free vancomycin (9 mg/kg) was only partially effective with the survival rate of 70–80% (n = 5–7) (Fig. 4b). In this study, the investigational peptide is highly selective for MRSA but was unable to deliver a drug to tissues infected with Gram-negative pathogen. Thus, the utility of such peptide will remain for narrow-spectrum antibiotics and this pathogen-specific targeting mechanism remained unclear.

Apart from the aforementioned antibiotic formulations, fluoroquinolone antibiotic also received much attention as a nanoformulation. Fluoroquinolones exert antimicrobial action by inhibiting either DNA gyrase or topoisomerase-II and thus, inhibiting replication and transcription of bacterial DNA [92]. In addition to antimicrobial effects, many drugs from the class are found to affect both cellular and humoral immunity by attenuating cytokine responses. Most fluoroquinolone derivatives superinduce in-vitro interleukin-2 synthesis but inhibit the synthesis of interleukin-1 and TNF-α [93]. The bacterial infection creates a distinctive surrounding around the lesions, termed as an infectious microenvironment (IME), comprised of a more acidic pH, presence of several bacterial enzymes, and activated blood vessels expressing several cell adhesion molecules such as intercellular adhesion molecule-1 (ICAM-1). Zhang et al. (2018) developed a novel mechanism-based bioresponsive nanoformulation that effectively delivers the antibiotic, ciprofloxacin and an anti-inflammatory agent, (2-[(aminocarbonyl) amino]-5-(4-fluorophenyl)-3-thiophenecarboxamide, TPCA-1). The multi-functional block copolymer used in the system is sensitive to pH or bacterial enzymes or both, which might trigger the dissembling of nanoparticles for drug release. The nanosystem was conjugated with ICAM-1 antibody, which targets the IME and localized nanoparticles to release the drug in response to IME. The benefits were evident with better survival in the CIP + TPCA-1-NPs-anti-ICAM-1-treatment group compared to the isotype control antibody-coated NPs (90% vs 50%; *p* < 0.05), while survival for free drug treatment and free ciprofloxacin were 40% and 30%, respectively (Fig. 4c). Free drug treatments showed 40% of mice survived for combined treatment of CIP + TPCA-1 and 30% for free CIP. This study has demonstrated the development of a novel delivery system that can target multiple targets in sepsis. First, application of ICAM-1 antibody leads to migration of the nanoparticles at the desired site, while inhibition of ICAM-1 by the antibody increased the specificity of neutrophil migration. Second, the use of bioresponsive nanoparticles, delivers and releases the drug to the infection site that helps to improve drug safety. Third, the nanoparticles were successfully loaded with both antibiotic and an anti-inflammatory agent, showing the possibility of multidrug loading in the delivery system [94]. Although, this report seems promising, it involved a complicated synthesis of a copolymer system; thus, the use of a simple synthetic approach will be valuable. A similar approach of targeting ICAM-1 was implemented by Yang et al. (2020) by using a broad-spectrum fluoroquinolone antibiotic, sparfloxacin, and an immunosuppressant agent, tacrolimus, to manage inflammatory response from bacterial infection. This nano delivery system combined the above mentioned hydrophobic drugs, loaded into PLGA NPs by a one-pot emulsion-based method, and surface functionalised with the γ3 peptide (NNQKIVNLKEKVAQLEA) to target inflammatory sites by binding with ICAM-1 [95]. The author proposed that this system, with an excellent broad-spectrum antibacterial activity, could effectively reduce inflammation and the immune response in mice with an acute lung infection. The survival rate of the group that received γ3-PLGA/S + T NPs was greater as compared to the disease model (75%, vs 12.5% respectively) (Fig. 4d). This reported method is a simpler alternative to the previously described nanosystem, which is more complicated [94].

Lipopolysaccharide (LPS) are components of the gram-negative bacterial outer membrane and can activate surface receptors, triggering the interaction of LPS with macrophages via the CD14 receptor with the help of TLR4. These interactions lead to the gradual release of a variety of proinflammatory cytokines, such as IL-8, IL1-β, and IL-6, causing a severe systemic inflammatory response. Targeting such interactions can alleviate sepsis progression. Mishra. P. R. (2011) reported on a chitosan-based lipid-nanoemulsion containing an ionic complex of ciprofloxacin with sodium deoxycholate (LE-CH-CFn-SDC) and evaluated it against a lipid nanoemulsion of ciprofloxacin with sodium deoxycholate (LE-CFn-SDC), as well as lipid nanoemulsion of free ciprofloxacin (LE-CFn). The findings from the study showed better outcomes in the order of LE-CH-CFn-SDC > LE-CFn-SDC > LE-CFn for studied parameters like loading efficiency, LPS induced mortality, LPS induced nitrite and TNF-α release, indicating the role of chitosan in LPS-macrophages interaction [96]. Further studies with this lipid nanoemulsion delivery system by the same group reported similar results [97]. Improved antimicrobial efficacy shown by LE-CH-CFn-SDC is attributed to the inherent antimicrobial activity of chitosan. Also, the interaction of LPS with CH induces a significant reduction in endotoxin release for CH coated formulations when compared to uncoated formulations. Another research group modified the formulation using different development methods, and have reported results in line with the aforementioned studies [98]. The formulation in this study was developed by a nano-precipitation technique comprising polycaprolactone (PCL) NPs for delivery of moxifloxacin (MOX) as antibiotic, and rutin (RT) as an antioxidant and anti-inflammatory agent. However, in this formulation, intracellular delivery of a lipophilic drug by a safer mean with improved therapeutic index is described, while the infection site-specificity was not well studied.

#### Nano formulations of antimicrobial peptides

Antimicrobial peptides (AMPs) have emerged as a new strategy and regarded as a promising solution for MDR bacterial infection due to their extremely rapid bacterial killing property [99]. Conventional antibiotics act on specific intracellular targets, whereas bacterial killing by AMPs is mediated through multiple mechanisms. The initial step is interaction with anionic bacterial membranes, through electrostatic interactions between the positively charged amino acids and the negatively charged cell surface, followed by hydrophobic interactions between the amphipathic domains of the peptide and the membrane phospholipids, leading to physical damage of the bacterial morphology [100]. The rapid bacterial killing kinetics through multimodal mechanisms reduces the risk of resistance development against these AMPs and may serve as a unique alternative against MDR bacterial infections.

Due to the unique features of AMPs and multimodal antibacterial mechanisms, much research interest is growing in combining AMPs with conventional antibiotics, based on the synergistic activity between these two. Further, AMPs can enhance the permeability of conjugated antibiotics, thereby enhancing the intracellular concentration of antibiotics. To validate this hypothesis of synergism, Fan et al. (2015) developed a liposome with a combined short antimicrobial peptide, S-thanatin (Ts), with an antibiotic agent, levofloxacin (Ts-LEV-LPs) [101]. The liposomes were prepared by mixing cholesterol which prevents self-contact and self-fusion, improving the stability of the liposome, viz. hydrogenated soybean phosphatidylcholine (HSPC) and aminopropyl-polyethyleneglycol (2000)-carbamyldistearoyl phosphatidylethanolamine (NHS-PEG2000-DSPE) to stabilize the liposome, and to mediate the liposome-bacterial membranes fusion. PEGylation prolongs drug circulation in the bloodstream. This prepared liposome is then loaded with levofloxacin, by the ammonium sulfate gradient method, with entrapment efficiency of ~ 76%. The concentration-specific combination of levofloxacin and Ts shows synergism with significant improvement of bacterial clearance in a sepsis mouse model, compared to levofloxacin liposomal formulation (P < 0.05), and MICs were 2–16 lower with Ts-LEV-LPs than the free drug in tested 17 clinical isolates of *K. pneumoniae*. The report proposed the underlying mechanism as (a) targeted delivery through the use of AMP, (b) hydrophobicity of liposomes enhancing drug entry, (c) increased drug update due to loss of structural integrity of the bacterial cytoplasmic membrane and active efflux failure, and (d) contact drug release using liposomes leading to more drug uptake than free drug formulation [101]. Thus, the report has shown that the use of a combination of nanotechnology and AMP, with an antibiotic agent, not only led to synergy but can be a feasible option in targeted drug delivery. However, the effect of divalent cations such as Ca++ and Mg++ stabilizes the bacterial outer membranes and may inhibit the interactions of an antimicrobial peptide with LPS [102]. Thus, extrapolating in vitro AMPs action on the bacterial membrane is difficult as in vitro studies do not always mimic in vivo studies.

**Fig. 5:**
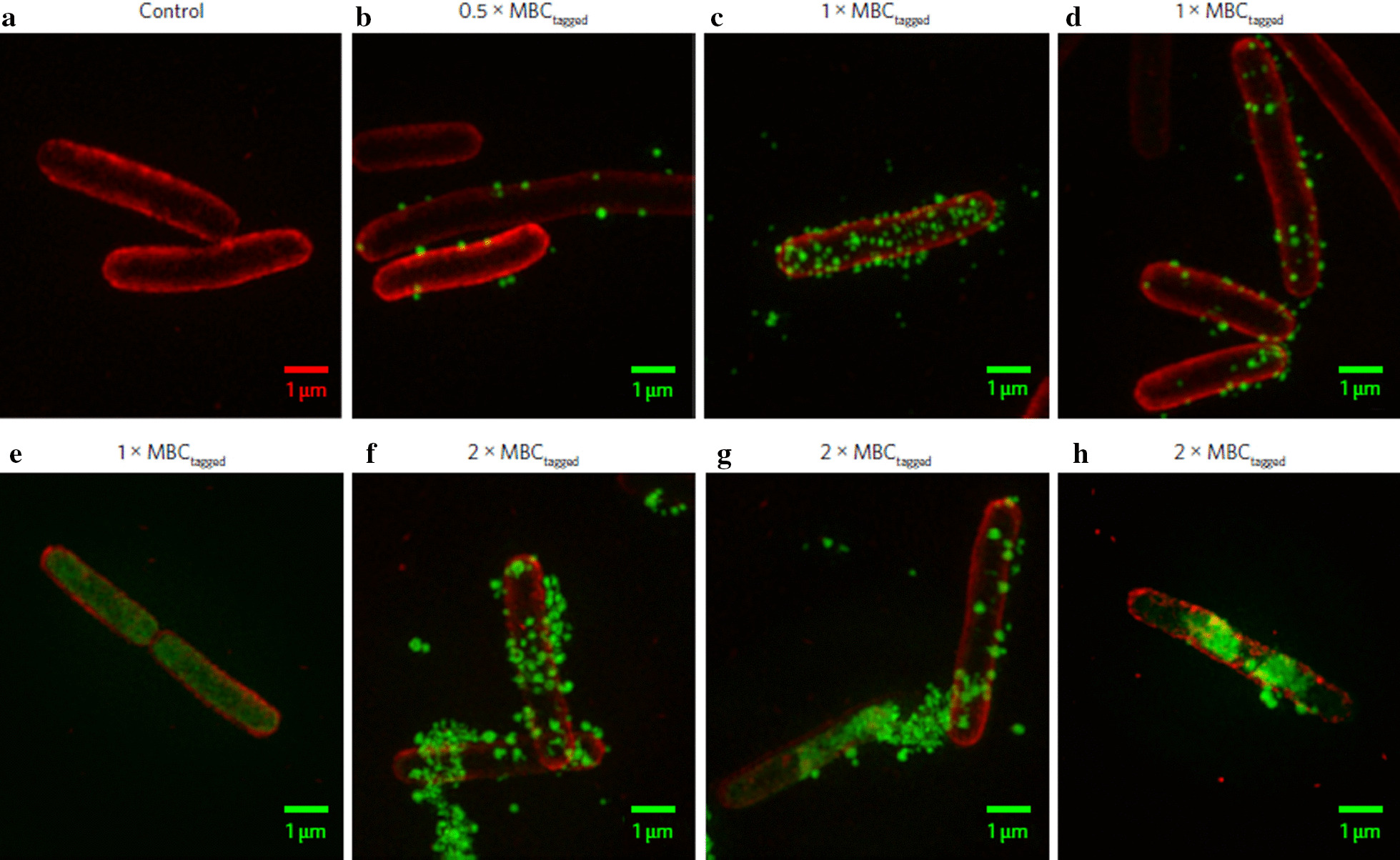
Fluorescence imaging studies using Optical Microscope eXperimental 3D-SIM images: Images of *E. coli* before (**a**) and after treatment with AF488-tagged SNAPP S16 (**b–h**). The *E. coli* cell membrane was stained with red and S16 with green in all images [100]

Application of AMPs in clinical settings are restricted due to their non-specificity and non-selectivity, leading to considerable toxicity in host cells [100]. Other common challenges associated with AMPs are degradation by bacterial proteases and the nonuniform pharmacokinetic profile. The application of nanotechnology can overcome these issues of cytotoxicity, degradation by proteases and efficiency at the target site [103]. By using a similar approach, Lam et al. (2016) recently discovered a new class of star-shaped, antimicrobial peptide polymer nanoparticles [100]. These star nanoparticles consisting of lysine and valine residues were termed ‘structurally nanoengineered antimicrobial peptide polymers’ (SNAPPs). In this study, two variable arm length star peptide polymer nanoparticles (S16 = 16 arm and S32 = 32 arm) were synthesized via ring-opening polymerization of α-amino acid N-carboxyanhydrides. Both these newly developed SNAPPs exhibit better antibacterial activity against clinically important gram-negative bacteria compared to gram-positive bacteria. Interestingly, the study also reported the significance of the star architecture in enhanced antimicrobial activity, as compared to linear analogues of the peptides, reflecting the importance of nanostructure in providing higher local charge concentration, facilitating strong ionic interactions with the bacterial membrane. The results of fluorescence imaging studies indicate the local accumulation of SNAPPs with electrostatic interactions on the outer membrane that destabilize and disrupt the cell membrane leading to cell death (Fig. 5a–h). At the same time, competitive inhibition assay with lipopolysaccharide (LPS) can explain SNAPPs selectivity towards gram-negative bacteria. Additionally, in vivo studies confirm the improved survival, better safety and efficacy against colistin-resistant strains of *A. baumannii*. Thus, SNAPPs may serve as a practical solution against resistant gram-negative bacterial infection.

In sepsis patients, besides resistance, the uncontrollable inflammatory response remains the most devastating factor. The pathophysiology of sepsis involves an initial inflammatory response followed by prolonged immunosuppression, causing immune paralysis, that leads to compromised host defence against pathogens [104]. There is clinical data to support the benefits of immune restoration therapies on survival and bacterial clearance from the host [105, 106]. This alternate therapeutic approach for restoring the immune function and eradicating the infection accompanied with nanotechnology, has been recently reported by Hou et al. (2020). In this study, vitamin lipid nanoparticles (VLNP) mediated adoptive transfer of macrophages delivering antimicrobial peptide linked cathepsin B (MACs) mRNA [107]. This newly designed delivery system comprised of MACs was shown to boost innate immunity, prevent bacterial immune evasion and eliminate resistant bacteria in immunocompromised septic mice model. VLNPs were prepared using water-soluble and fat-soluble vitamins (Vitamin -B3, B7, C, D and Vitamin-E). V_C_LNPs undergo caveolae-mediated endocytosis with 20-fold more effective mRNA delivery than other four VLNPs. The V_C_LNPs was also combined with bone marrow-derived macrophages and found effective against monomicrobial induced sepsis as well as polymicrobial induced sepsis in immunocompromised septic mice. Although the application of autologous MACs is critical at present but have the potential for clinical therapeutic application in the near future.

#### Other antimicrobial nanoformulations

In response to increasing bacterial resistance to conventional antibiotic formulations, in addition to the aforementioned nano-antibiotic formulations and AMPs, alternative antimicrobials are of prime importance. The alternative strategy also includes exploration of antimicrobial compounds from ‘natural’ origins, as well as metal, and metal oxide nanoparticles. Amongst compounds of natural origin, in particular, terpenoids and aromatic compounds, occurring in essential oils, are a potential reservoir for antimicrobial compounds. However, potent antimicrobial activity of aromatic compounds, over terpenoids, is due to the presence of certain phenolic, aldehyde and alkene compounds. Many of these compounds like, carvacrol, eugenol, cinnamaldehyde and β-caryophyllene are remarkably shown to possess broad-spectrum antibacterial activity, and their combinations were proven to be synergistic [108]. The most common challenge with these essential oils is their highly volatile nature and poor bioavailability because of hydrophobicity (low water solubility). To overcome these challenges, the development of lipidic nanocapsules (LNCs), encapsulating such lipophilic active compounds, was reported by Montagu et al. (2014). This is a very early report of a nanoformulation with essential oils of spices and herbs such as origanum, cinnamon, and clove. They have the most potent antibacterial properties because of their major components which are carvacrol, cinnamaldehyde and eugenol, respectively. The developed nanoformulation was unable to show better antimicrobial activity in vivo, due to the need for LNCs surface modification and functionalization to target bacterial cells. The functionalization with CH or other cationic molecules may modulate the LNCs interaction with negatively charged bacterial cell membranes and thus effectively deliver antimicrobials [109].

Amongst other alternatives nanomaterials, metal and metal oxide nanoparticles exhibit notable broad-spectrum antimicrobial activities and are often referred to as “nanoantibiotics”. There are reports that bacterial resistance development against nanomaterials is less likely, making this an attractive option for development. Several mechanisms have been proposed through which nanomaterials exhibit their bactericidal activity, involving disruption of the bacterial cell membrane, denaturation of protein and DNA damage and disruption of the respiratory chain. The silver NPs are extensively studied in various in vitro experiments and have proven antimicrobial property against different pathogens; however, their application for in vivo models remains a concern due to safety. Kuthati et al. (2015) have reported the pH sensitive release of silver-indole‑3 acetic acid complexes from mesoporous silica nanoparticles. This newly reported pH-sensitive hydrazone bond mediated metal complex-conjugated NPs shown enhanced antimicrobial activity against multidrug resistant clinical isolates as well as inhibit biofilm formation of *E. coli, B. subtilis, S. aureus*, and *S. epidermidis* [110]. The another study explored the modes of transformation of carbon membrane packaged Ag nanoparticles (Ag-C), namely, the packaging, the activation, and the deactivation, triggered by different concentrations of PBS solution, were comprehensively profiled for in vitro activities [111]. However, there is uncertainty regarding antibacterial activities of Au-NPs in the physiological environment or in vivo models. A study was later conducted on the possibility of controlling the metabolism of Au-NPs in the animal model [112]. Zeta potential of Au-NPs with different salts, such as PBS, NaCl, K_2_HPO_4_, and KCl, were measured and found to be lowest with NaCl and reduced cytotoxicity of Au-NPs was observed with the addition of 4 × NaCl (group according to dilution). There was no evidence for accumulation in metabolic organs when evaluated in septic mice models. In this study, though mean survival time was found to be improved, the survival rate was not improved and mechanism yet to be elaborated.

Other metals and metal oxide NPs such as zinc oxide, copper oxide, magnesium oxide and titanium dioxide have been evaluated for their in vitro antimicrobial activity [36]. Most recent report by Kankala et al. (2020) shown copper-doped mesoporous silica nanoparticles (Cu-MSNs) establishing pH-responsive coordination interactions with the tetracycline (TET) which improves loading efficiency and TET release in the acidic pH. Additional coating Cu-MSNs with ultrasmall silver nanoparticles-stabilized polyethyleneimine (PEI-SNP) stimulates the production of toxic free radicals responsible for disrupting the bacterial components. Thus, the reported MSN-based trio-hybrids (PEI-SNP@Cu-MSN-TET) synergistically exhibited profound antibacterial activity against resistant *E. coli* strain [88]. The similar trio-nanohybrid approach has been reported with synergistic antibacterial effects by combining natural antimicrobial agent, curcumin, with above reported Cu-MSNs and SNP (Cur-Cu-MSN-SNP). However, this trio-nanohybrids shown the application of emerging photodynamic inactivation technique as an alternative to antibiotics against resistant *E. coli* [113]*.* Interestingly, the selectivity of most NPs to Gram-negative pathogens over Gram-positive pathogens is common, which may be attributed to a thicker cell wall in Gram-positive pathogens [114]. Apart from these observations, few studies have reported species-specific and strain-specific variation, which can be useful in specific pathogen targeting strategies. Further, functionalized NPs employing amino group, sugars and carboxylic groups when evaluated against *E. coli* and *S. aureus,* and functionalization of amino and carboxy group, leads to significant improvement in antimicrobial and antibiofilm activity only against *E. coli* but not against *S. aureus* [115]. This sparsity in selectivity remained unanswered and future studies may reveal the underlying mechanism.

In a situation where barely any novel antibiotic drugs are close to approval for clinical use, there is a need to use existing antibiotics with caution. Efforts have been made to develop various nanoformulations of antimicrobials. An overview of nanoformulations, including nanosystems used in various reports, and their characterization is summarized in Table 2. It is evident from the data that a broad range of pathogens, including MDR strains, have been evaluated using in vitro and in vivo sepsis models, and the use of available antibiotics through nanoscale conversion, has some benefits pertaining to efficacy and may overcome microbial resistance. Thus, advantages of the nano-sized drugs in their biodistribution and nanotechnology-based targeted delivery has unexplored potential in sepsis management.

**Table 2:** Overview of key features of various antimicrobial nanoformulations

Nanosystem		Characterization			Target pathogens		Key findings/ outcomes	References
Active	Nanocarriers	Zeta-potential	Shape / Size	LC/ EE (%)	In vitro	In vivo (Model)		
*Antibiotic nanoformulations*								
Meropenem	CH with tripolyphosphate (TPP) crosslinker	17.1 ± 2.3 to19.8 ± 2.6 mV	Spherical 261.8 ± 37.5 nm	EE = 71.5–76.3	*E. coli* *K.pneumoniae* *MRSA* *MSSA*	*K. pneumoniae* (Systemic infection/ sepsis in rat)	Two-fold lower MIC Improved survival	[91]
Vancomycin (VCM)	Peptide (CARG)-conjugated Porous silicon nanoparticles	–	~ 180 nm	LC = 12	*S. aureus* *MRSA* *P. aeruginosa*	*S. aureus* (Lung infection model in mice)	Targeted binding to *S. aureus* by CARG Reduced systemic dose in vivo	[89]
Ciprofloxacin and TPCA‐1	Block copolymer Biotin-PEGb-PAE (-g-PEG-b-DSPE)-b-PEG-Biotin	− 6.81 mV at pH 7.4 + 7.35 mV at pH 6.5	Spherical 120 nm	LC = 9.2 ± 0.2 EE = 53.7 ± 1.3	*P. aeruginosa*	*P. aeruginosa* (Acute lung bacterial infection in mouse)	Targeted on-demand delivery in response to IMEs Enhanced therapeutic efficacy and survival	[94]
Sparfloxacin and Tacrolimus	γ3 peptide grafted on poly (lactide-co-glycolide acid) (PLGA)	− 40 mV	Spherical 183.7 ± 9.4 nm	(SFX 5 mg/ml) EE = 84.7 (SFX) 85.6 (TAC)	*P. aeruginosa* *S. aureus*	*P. aeruginosa* (Acute lung infection in mice)	Increased binding to inflamed cells by γ3 peptide Decreased inflammation and immune response in vivo	[95]
Ciprofloxacin	Lipid emulsion of chitosan and sodium deoxycholate	+ 28.2 ± 2	225 to 325 nm	EE = 93.7 ± 2.3	*E. coli*	*E-coli* (Peritonitis or abdominal sepsis model in rats)	Decreased TNF-α and NO production Improved survival	[97]
Moxifloxacin and Rutin	Poly-caprolactone	− 22.63 ± 0.55 mV	173.63 ± 3.90 nm	LC = 7.49 ± 0.31 EE = 72.64 ± 1.06	*E. coli*	–	Suppressed LPS released from bacteria	[98]
*Antimicrobial peptide and other nanoformulations*								
S-thanatin with levofloxacin	Liposome prepared with HSPC, CHO and Ts-PEG2000-DSPE	+ 5.3	152.5 ± 3.2	LEV EE = ∼76	MDR *K. pneumonia*	MDR *K. pneumonia* (Septic shock model in mice)	2–16-dilution lower MIC than free drug Improved efficacy on bacteria clearance	[101]
Structurally nanoengineered antimicrobial peptide polymers (SNAPPs)	Poly(amido amine)	–	Star-shaped S16 = 7.8 ± 1.2 nm S32 = 7.5 ± 1.6 nm	–	Streptococcus mutans *S. aureus* *E. coli* *P. aeruginosa* *K. pneumoniae* *A. baumannii*	*A. baumannii* (Peritonitis model in mouse)	Superior antibacterial activity against colistin-resistant and MDR pathogens Higher therapeutic indices	[100]
Antimicrobial peptide and cathepsin B (AMP-CatB) mRNA	Vitamin lipid nanoparticles (VLNPs)	~ 22 mV	~ 140 nm	EE = ~ 90	MDR *S. aureus*	MDR *S. aureus* MDR *E. coli* (MDR bacteria-induced sepsis in mice with immune-suppression)	Demonstrated adoptive transfer of MACs Improved recovery of immune-compromised septic mice	[107]
Mixtures of Carvacrol and Eugenol, Cinnamaldehyde and/or β-Caryophyllene	Lipidic nanocapsules	− 16 ± 2 mV	66 ± 4 nm	LC = 20 EE = 49	*A. baumannii*	*A. baumannii* (Pathogen induced sepsis in mice)	Synergistic antimicrobial activities in combination Improved survival	[109]
Silver based nanoparticles	Carbon quantum dots	− 52.12 ± 6.81 mV	13.23 ± 4.03 nm	–	*S. aureus* *E. coli* *P. aeruginosa*	(High-grade sepsis in mice by Cecal ligation and puncture)	Ameliorated inflammation in the heart, liver, spleen, lungs, and kidney	[112]

### Nanotechnology and anti-inflammatory agents

Sepsis pathophysiology involves both cytokine-mediated inflammation counterbalanced by anti-inflammatory responses, providing numerous potential targets to ameliorate sepsis [116]. The innate immune response activation is facilitated by toll-like receptors (TLRs). TLRs are activated after forming a complex with PAMPs, such as LPS in Gram-negative pathogens or lipoteichoic acid (LTA) in Gram-positive pathogens. Further downstream signalling process recruits proinflammatory intermediates, like mitogen-activated protein kinases (MAPKs), Janus kinases (JAK) and nuclear factor κ (kappa)-light-chain-enhancer of activated B cells (NF-κB) [117]. Thereafter nuclear localization of these proinflammatory intermediators initiates gene expression and activation of cytokines including TNF-α, IL-1/12/18 and type-1 IFNs triggering further cytokine and chemokine production such as IL-6/8, IFN-γ culminating into a cytokine storm. However, TNF-α regulates most of the downstream cascades and widely accepted role of IL-1 in inflammation, thus getting more focus as a target for therapeutic development [118]. Nanotechnological applications in anti-inflammatory treatment are presented here under two broad categories; those are mediated through interaction with TLRs or reported as cytokines inhibitors.

Different PAMPs are recognized by specific TLRs and are vital in infectious diseases while TLR2 and TLR4 are critical to the sepsis. TLR4 can bind to LPS and recognizes Gram-negative bacteria [35]. This is a potential target for future therapeutic strategies in the management of inflammation in infectious diseases and more specifically, inflammation in sepsis due to bacterial infection. Based on the ability of several natural compounds, e.g. lipid A, to modulate TLR4 activity and using molecular diversity-oriented strategies and structure–activity relationship (SAR) studies, Lavado et al. (2014) developed a cationic glycolipid-coated GNP system, interacting with CD14 and MD-2 receptors, to modulate TLR-4 activity [119]. Further to potentiate its action, glucose and trehalose based cationic glycolipids coated on gold NPs by surface adsorption, was prepared. This newly developed nanosystem was able to inhibit LPS activity and inhibit the LPS-induced TLR4-MD2 activation in vitro*,* similar to that of synthetic antagonist eritoran. Although compounds adsorbed on gold nanoparticles were active as the TLR4 antagonist in vitro*,* it shows high toxicity in animal models. Other studies on peptide-gold nanoparticle hybrids show potent TLR inhibition in vitro [120, 121], toxicities due to non-degradable core or bioaccumulation of gold NPs as reported in other studies limit their application for in vivo studies [122, 123].

**Fig. 6:**
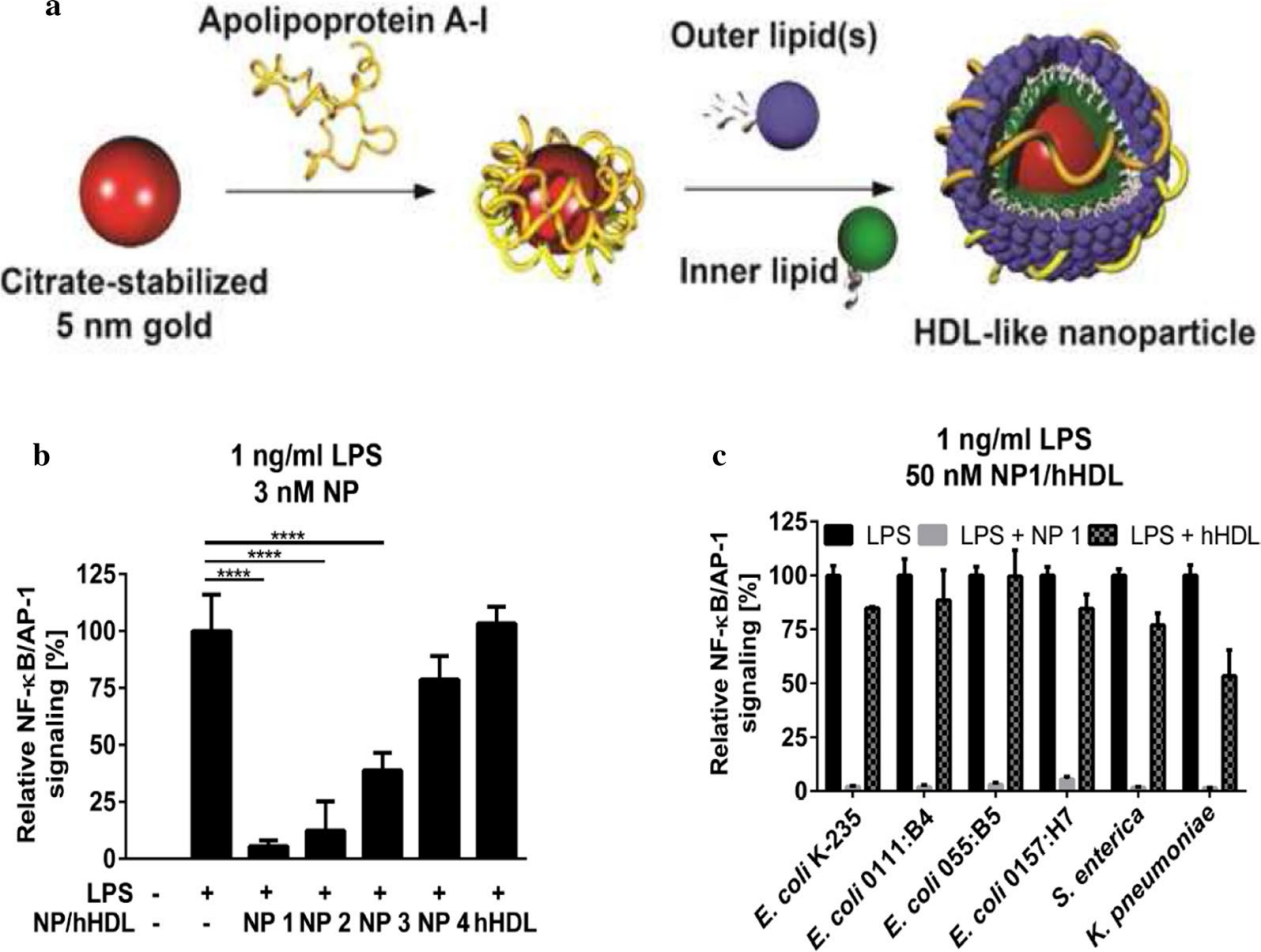
HDL-like NP effect on suppressing TLR4 signalling. **a** General scheme for the synthesis of HDL-like NP. **b** Effect of different HDL-like NP and human HDL (hHDL) on inflammatory response when reporter cells treated with 1 ng/ml LPS from *E. coli*. **c** Inflammatory response of NP 1 or hHDL and LPS derived from the different bacterial species as indicated (****p ≤ 0.0001) [124]

The other approaches in LPS sequestering were demonstrated with the help of binding with high-density lipoprotein (HDL) like nanoparticles, in a study by Foit and Thaxton (2016). HDL is involved in detoxification of LPS and a similar phenomenon was implemented in the development of the nanosystem. Citrate stabilized colloidal gold nanoparticle (Au NP) was used as a scaffold and was functionalized with apolipoprotein A1 (apo A-I). The apo A-I-gold complex mimics human HDL, due to presence of apo A-I., and the resultant complex was then decorated with a varying constituent component of the conjugates phospholipid bilayer and schematic presentation is given in Fig. 6a. All the newly reported NPs were more potent than human HDL (hHDL) to reduce the LPS-mediated inflammatory response (Fig. 6b). The most potent HDL-like NP, construct 1, made up of lipids PDP PE 16:0, cardiolipin and 18:2 PG, led to the largest decrease in NF-kB/AP-1 signalling induced by LPS-derived from the different bacterial species [124] (Fig. 6c). Thus, the author proposed the biomimetic approach using HDL with inherent benefits of scavenging and neutralizing the LPS toxin from the different bacterial species, non-toxicities and non-inducers of inflammation.

Contrary to inhibition of proinflammatory cytokines, agents that promote the production of anti-inflammatory cytokines such as IL-4, IL-10, and IL-13 can serve as a novel and efficient strategy in sepsis management. Xu et al. (2019) have recently reported on activated macrophage autophagy on increasing anti-inflammatory cytokine, IL-10 by superparamagnetic iron oxide nanoparticles (SPIONs) of γ-Fe2O3 nanoparticles. SPIONs-induced autophagy in macrophages is prompted through activation of the Cav1-Notch1/HES1 signalling pathway [125]. The IL-10 expression has been shown to be augmented with the treatment of Siglec-targeting platforms consisting of poly(lactic-co-glycolic acid) nanoparticles decorated with a natural Siglec ligand, di(α2 → 8) N-acetylneuraminic acid (α2,8 NANA-NP) [126]. Murine sialic acid-binding immunoglobulin-like lectin-E (Siglec-E) and its orthologs, Siglec-7 and Siglec-9, are critical negative regulators of acute inflammatory responses and are capable of inhibiting TLR signalling.

However, other mechanisms involved in the anti-inflammatory activity of various nanoparticle-based delivery has been reported by another research group. The most common anti-inflammatory mechanism exhibited by nanosystems is mediated through TLR4-related NF-κB pathway. Astragalus polysaccharide (APS) nanoparticles, synthesized with chitosan derivatives (CS/TPP) as the drug carrier, was shown to be effective in cell-based studies, as well as cecal ligation and puncture-induced mice model of sepsis [127]. Inhibition of the same pathway led to a reduction in proinflammatory cytokines TNF-α, IL-1β, IL-6, by intravenous administration of cerium oxide nanoparticles in lipopolysaccharide (LPS) sepsis model in Sprague Dawley rats [128]. Wang et al. (2015) reported strong inhibitory effects of curcumin (Cur) loaded solid lipid nanoparticles (Cur-SLNs) on pro-inflammatory cytokines through nuclear factor (NF-kB) signalling [129].

Although clinical application of anti-inflammatory therapy remains controversial, it has the potential to ameliorate sepsis progression due to the central role of the pro-inflammatory markers in sepsis pathophysiology. Unfortunately, clinical studies with anti-inflammatory agents were unable to reproduce significant efficacy based on preclinical studies. However, such data variability can be explained by heterogeneity amongst septic patients which cannot be mimicked by existing preclinical models. Currently, increasing mechanistic studies at the biomolecular level and their impact of pathophysiological pathways in sepsis will provide more insights for tailoring approaches in anti-inflammatory therapy. In Table 3, different anti-inflammatory mechanisms are summarized with their impact on physiological processes.

**Table 3:** Different anti-inflammatory mechanisms of action (MOA) exhibited by various nanoformulations

Nanosystem	Size	MOA	Key findings	References
Peptide decorated-gold nanoparticles	13–14 nm	Modulate endosomal pH Blockade of endosomal acidification Inhibits downstream TLR4 signalling pathways, leading to the reduction of NF-kB, IRF3 and MAPK activation	Improved the disease activity index Ameliorated colonic inflammation in vivo	[119]
Astragalus polysaccharide (APS) NP	105–115 nm	Inhibited the activation of TLR4/ NF-κB pathway	Decreased myocardial inflammatory cytokine expression	[127]
High-density lipoprotein-like nanoparticles	Similar to hHDL	LPS toxin scavenging and neutralizing	Decreased TLR4 signalling Inhibited inflammatory response to LPS	[124]
Curcumin-loaded solid lipid nanoparticles	40–80 nm	Suppressions of NF-kB activation and IkBa degradation levels	Decreased expression of pro-inflammatory cytokines (IL-6, TNF-α, and IL-1b)	[129]
Cerium oxide nanoparticles	–	Decreased transcriptional action of ROS, iNOS, COX-2, and nuclear factor-kappa light chain, the triggered B cells (NF-kB)	Decreased hepatic damage, serum cytokines/ chemokines, and swelling indicators in vivo	[128]
Trehalose- and glucose-derived glycoamphiphiles incorporated in Au NP	–	Interference with TLR4 activation and signalling in vitro and in vivo	Inhibited LPS-triggered IL-6 production in mice	[119]

### Nanotechnology-based antioxidants

In response to the pathogenic invasion, the host initiates defence mechanisms, including inflammation, where activated inflammatory and immune cells also attenuate reactive oxygen species (ROS) production facilitating clearance of pathogens, but excess ROS release, as seen in sepsis, leads to oxidative stress [130]. Oxidative stress represents an imbalance between the levels of oxidants or reactive oxygen species (ROS) and endogenous antioxidants. Proinflammatory markers induce the production of ROS, including the potent hydroxyl radical, nitric oxide, superoxide, hydrogen peroxide, peroxynitrite and hypochlorous acid that participate in sepsis pathogenesis [131]. They have a profound effect on endothelial cells, thus promoting vascular permeability, aggravating hypotension and decreasing the colloid osmotic pressure of the plasma. ROS also affects oxygen consumption by septic cells leading to “cytopathic hypoxia,” which accelerates the process causing multiple organ failure [132]. Furthermore, hyper-inflammation and persistent oxidative stress trigger changes in the mitochondria causing mitochondrial dysfunction, which contributes to excessive production of ROS and cellular energetic failure. The overproduction of mitochondrial ROS (mtROS) further acts as signalling molecules that trigger the upregulation of inflammatory cytokine production [133]. Altogether hyper-inflammation, oxidative stress and mitochondrial dysfunction are responsible for poor sepsis outcomes. ROS also regulates the release of transcription factors such as NF-κB, nuclear factor-like 2 (Nrf-2) and activator protein-1 (AP-1), which after nuclear localization, induces activation of several genes that express inflammatory proteins, and hence targeting ROS could be an attractive target in sepsis management [130, 131].

Several reports on antioxidant compounds including, vitamins, minerals and endogenous free radical scavengers, such as melatonin, have shown benefits in ameliorating oxidative stress and sepsis outcomes, in preclinical and clinical studies [131]. Use of melatonin is of significant interest due to its protective effect, through inhibition of NF-κB and NLRP3 inflammasome activation but possesses limitations in therapeutic applications due to its short half-life (t_*1/2*_ < 30 min) and low bioavailability. Volti et al. (2012) evaluated the systemic therapeutic approach of two different melatonin-loaded nanocarriers, prepared from poly (d, l-lactideco-glycolide), (PLGA [NP-A]), and diblock poly [(d, l-lactide-coglycolide)-co-PEG], (PLGA-PEG [NP-B]). For NP-B, the surface modification with poly (ethylene glycol) (PEG), provides hydrophilic and non-ionic characters that prevent macrophagic uptake, thus leading to longer circulation times. An experimental animal model of sepsis was used as a model to measure heme oxygenase-1 (HO-1), which is ubiquitous in all organs, with increased expression in response to oxidative stress. PLGA-PEG NPs were smaller (83.80 + 1.10 nm), spherical, and are homogeneous with smooth surfaces, and have higher encapsulation efficiency for melatonin. NP-B melatonin NP formulations significantly affect HO-1 protein and decrease linoleic acid hyperperoxide (LOOH) levels in the heart, lung and liver, when compared with NP-A, free melatonin or vehicle. Thus the report confirmed the profound antioxidant benefits of melatonin and its application in sepsis with specific drug delivery systems [134].

Importantly, besides systemic disturbances induced by oxidative stress, excessive production of ROS in sepsis have a significant impact on hepatic cells, causing sepsis-induced acute liver injury. During the early progression of sepsis, neutrophils are recruited to the liver and their antibacterial action produces ROS, which induces production of proinflammatory cytokines and chemokines. However, this overproduction of ROS can lead to cascades that cause cellular toxicity, apoptosis or necrosis in the septic liver [135]. Thus, maximum drug accumulation at the site of injury, such as the liver may result in better outcomes than systemic distribution. This could explain the failure of antioxidant drugs in clinical trials, despite promising results in preclinical studies. Stimuli-responsive drug delivery systems serve as on-demand delivery and targeted release in response to specific microenvironments. Chen et al. (2017) explored ROS-responsive polymeric spherical NPs of melatonin, formed using deblock copolymers of poly(ethylene glycol) (PEG) and poly(propylene sulfide) (PPS) (127 nm), which undergo an oxidative conversion enabling on-demand delivery of the drug for alleviating sepsis-induced liver injury [136]. Additionally, intraperitoneally administered drug in this study, undergoes the first-pass metabolism and thus, the maximum amount of drug gets deposited in the liver and additionally, the nanoscale size helps accumulation in mitochondria of hepatic cells generating maximum ROS load. The study result reveals the sensitivity of Mel-loaded PPS-NPs to ROS. Under the conditions of oxidative stress, upon exposure to ROS, hydrophobic sulfide moieties of the polymer converted to more-hydrophilic sulfoxides and sulfones, accelerating disassembly of the NPs. The findings of this study also show superior outcomes, compared to previously described reports of Volti et al. (2012), in terms of ROS-responsive drug delivery systems with controlled melatonin release at target sites that overproduce ROS. Thus, the proposed on-demand delivery system has the potential to deliver antioxidant agents to tissues or sites affected by ROS.

An important consideration on the role of nanotechnology in safety enhancement of antioxidants, without compromising efficacy, is reported by Soh et al. (2017). In this study, ceria-zirconia nanoparticles (CZ NPs, 2 nm) were proposed as potential antioxidant nanomedicines for treating ROS-related inflammatory diseases. Ceria nanoparticles attained importance due to its potential antioxidant property; nevertheless, safety concerns limit the dosage used in various applications. Their free radicle scavenging activity depends on particle size and the atomic ratio of surface Ce^3+^ to Ce^4+^. Zirconia (Zr^4+^) is incorporated in the formulation to control the Ce^3+^/Ce^4+^ ratio and the rate of conversion between two oxidation states. These CZ-NPs were initially synthesized with varying ratios and were studied in vitro. The formulation of CZ NPs (Ce_0.7_Zr_0.3_O_2_) exerts a powerful antioxidant effect in aqueous media, in vitro studies as well as two in vivo models of sepsis, namely, LPS-induced endotoxemia rat model and CLP induced bacteremia mouse model. With significant antioxidant properties, CZ NPs showed 100% survival in LPS induced sepsis model, and 2.5-fold more survival benefit in CLP induced bacteremia mouse model *vs* control group. However, in this study, CZ NPs were administered in LPS induced sepsis model simultaneously, whereas, in clinical settings, treatment usually commences after the onset of proinflammatory mediators.

### Nanotechnology-based extracorporeal blood cleaning

The occurrence of microbial toxins (released by both live and dead pathogens) or debris, probably caused by antibiotic treatment in the bloodstream, triggers systemic inflammation and causes multi-organ failure, septic shock and death. Due to high incidences of negative blood culture reports (~ 70%) in septic patients, and failure of antibiotics due to MDR infections; removing the source of infection from the extracorporeal circuit is getting more attention amongst researchers and clinicians. The existing approved extracorporeal blood filtration devices, such as hemofiltration devices, can be modified or functionalized with substances capturing different pathogens or their products and can be used for removal of pathogens. Extracorporeal blood cleaning approaches, integrated with nanotechnology, as a promising adjuvant therapy in sepsis, is discussed below.

Magnetic particle-based, blood cleansing methods, are most conventionally used due to the ability to separate cellular contents from a heterogeneous mixture, such as whole blood. Kang et al. (2015) predicted the benefit of considerably higher collision rate constant of diffusion (k_d_) with mathematic modelling [137]. An initial study, using this magnetic extraction-based extracorporeal removal method, was reported by Herrrman et al. (2010), based on direct injection of stable nanomagnets into whole blood, which efficiently removed low and high molecular-weight compounds [138]. Carbon-coated iron carbide (Fe_3_C) were used to prepare magnetic reagents which are further functionalized, based on the target compound to be removed from whole blood, such as ethylenediaminetetraacetic acid (EDTA) as chelators for heavy metal removal and whole antibody/fragments for removal of drug (digoxin) or proteins (IL-6). The limitation with this approach is the mandate of early information of the detoxification target or source of infection. Due to time-consuming pathogenic identification and the high cost associated with antibody-based methods, the feasibility of this method in clinical settings are restricted. To address these challenges, Lee et al. (2014) developed magnetic nanoparticles (MNPs) modified with zinc-coordinated bis (dipicolylamine) (bis-Zn-DPA) [139] that selectively and rapidly separate endotoxins, as well as pathogens, from whole blood. As bis-Zn-DPA specifically forms bonds with anionic phospholipids of Gram-positive and Gram-negative bacterial cells, their affinity toward normal mammalian is negligible. Based on these findings, the ease of synthesis and conjugation with rapid binding kinetics, compared to antibody-based approaches, enable its clinical application.

Moreover, other approaches in addressing the issues discussed above, include Kang et al. (2014), who reported the application of a combination of magnetic nanobeads, coated with an engineered human opsonin-mannose-binding lectin (MBL) and magnetic FcMBL-coated beads, for effective blood cleansing [140]. This easily manufactured MBL binds to a wide variety of pathogens and is effective in removing pathogens and endotoxins simultaneously. The broad-spectrum binding ability of MBL opsonin offers the dual advantage of treating systemic blood infections, without culture reports, by rapid whole blood cleaning and preventing sepsis progression.

### Nanotechnology-based antitoxins

Toxins that are released due to bacterial lysis, during antibiotic therapy, is an attack mechanism exhibited by a variety of organisms, including bacteria, promoting their survival. Toxin receptors are present on specific cells in the host but not others; thus, hampering physiological processes. Toxins can act through different mechanisms, including structural disturbances due to interaction with the membrane, perturbation of membrane-associated processes and signalling or interfering with an intracellular process such as protein synthesis [141]. The importance of toxins in the protection of pathogens from immune defences, supports detoxification approaches which can be a promising and effective alternative adjuvant therapies to existing therapeutic options. Several methods have been reported for endotoxin removal, including adsorption, filtration, ionic interaction and phase separation [142]. Conventional strategies have relied heavily on structure-based neutralization strategies, such as antibodies; however, variations in structural motifs and the diversity of toxins secreted across bacterial genus, species, and strain, presents a challenge with low reduction efficiency. Other challenges include the requirement of simultaneous administration of multiple formulations, and the toxicity of endotoxin neutralizing agents, limiting their clinical utility [142, 143]. The application of nanotechnology-based solutions can overcome these challenges due to the inherent properties of nanoparticles, such as more extended circulation and the potential for multivalent toxin interaction.

Many bacterial pathogens secrete cholesterol-dependent cytolysins (CDCs), α-hemolysin or bacterial phospholipase C, that act as pore-forming toxins, damaging the host cell membrane and have a crucial role in the progression of infectious diseases. Bacterial toxins have specificity for animal cells as they target cholesterol and sphingomyelin. Henry et al. (2015) explored engineered artificial liposomes, used as decoy targets, to sequester bacterial membrane-damaging toxins to compete with host cells for toxin binding. Liposomes, composed of sphingomyelin in combination with an artificially high concentration of cholesterol, effectively bind CDCs, phospholipase C and α-hemolysin while in combination with sphingomyelin liposomes (sphingomyelin-only liposomes), efficiently sequestering toxins released by a variety of staphylococcal and streptococcal pathogens. Enhanced binding of liposomes to bacterial toxins, protect human epithelial and endothelial cells that prevent bacterial invasion, protecting the innate immune system from the toxin-induced lysis, thus, leading to resolution of the infection. This phenomenon was evident with reduced cytokine (TNF-α) levels and bacterial load in an animal model. Further, treatment with monotherapy of low dose and a high dose of vancomycin (12.5 mg/kg/injection, 100 mg/kg/injection, respectively) was unable to afford protection from systemic infection, whereas the combination of liposomal toxin-sequestration and antibiotic treatment, provided complete protection. Thus, it is evident that a combination of liposomes with antibiotic treatment for susceptible pathogens, can improve the outcomes in patients, while for the treatment of antibiotic-resistant bacterial pathogens, neutralization of bacterial toxins protects the host immune system, leading to a reduction in bacterial load. Notably, a lack of direct interaction between bacteria and liposomes does not pose any risk for the emergence of drug-resistant pathogens. However, this study evaluated liposomal formulation in Gram-positive pathogens, its ability to sequester toxins secreted by Gram-negative pathogens are yet to be studied.

**Fig. 7:**
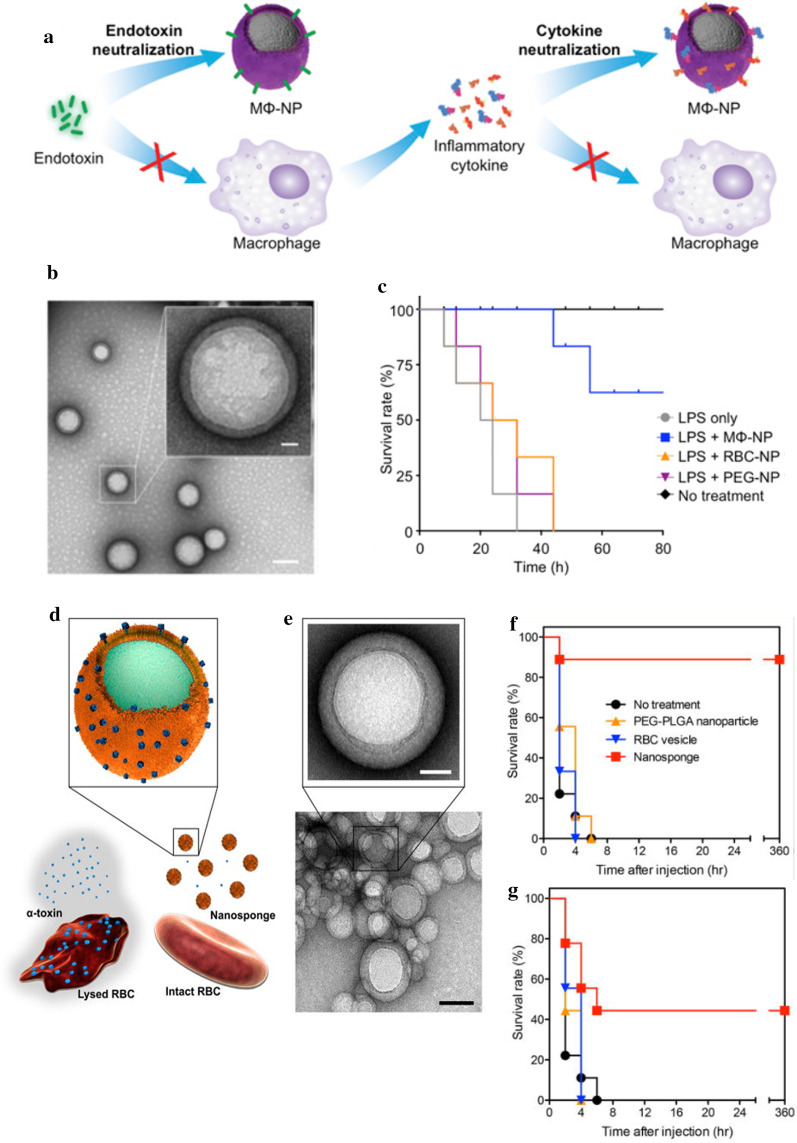
Formulation of macrophage membrane-coated NP (MΦ-NPs), **a** Schematic representation of the mechanism of endotoxin and proinflammatory cytokine neutralization. **b** Characterization by DSC, **c** Effect on survival based on in vivo studies [144]. **d** Biomimetic nanosponges and their mechanism of neutralizing pore-forming toxins **e** TEM visualization of nanosponges mixed with α-toxin, Survival rates of mice over 15 days after IV administration of blank or treatment agents 2 min either before (**f**) or after (**g**) the toxin injection [143]

Another group of researchers have reported that macrophage biomimetic nanoparticles (MΦ-NPs), developed by wrapping polymeric cores with cell membrane-derived from macrophages, bind and neutralize endotoxins [144]. MΦ-NPs bind to endotoxins, due to identical antigenic exterior to the source macrophage cells, and simultaneously act as decoys to bind to cytokines, inhibiting progression to cytokine storm, and thus having significant potential in sepsis management (Fig. 7a, b). In a mouse *E. coli* bacteremia model, treatment with MΦ-NPs also conferred a significant survival advantage (Fig. 7c). Biomimetic toxin nanosponge approach proposed by Hu et al. (2013), constructed with a polymeric core, that stabilizes the RBC membrane shell and ensures prolonged systemic circulation was wrapped in natural RBC bilayer membranes, thus absorbing a wide range of structurally different pore-forming toxins. The general mechanism involved in neutralizing pore-forming toxins, and characterization by TEM is given in Fig. 7d, e. Nanosponge antitoxin efficacy was evaluated in vivo by administering a lethal dose of 75 μg/kg α-toxin through the tail vein. Nanosponges were injected either 2 min before or 2 min after the toxin injection. In the nanosponge pre-inoculation group, the mortality rate was 11% vs 100% in control formulations group at 6 h (p < 0.0001, Fig. 7f). In the post-inoculation treatment groups, survival benefits were significantly higher than control formulations (44% survival, p = 0.0091, Fig. 7g). Extending survival benefits past 6 h to 15 days, suggests the detoxification of α-toxin, rather than its toxicity delayed [143]. However, varied susceptibility to the α-toxins by nanosponges, prepared from human and mouse RBCs, underscores the significance of membrane-toxin affinity for interactions with toxins. Wang et al. (2015) reported surface functionalization with acrylamide as the monomer and poly(ethylene glycol) dimethacrylate (PEGDMA) as the cross-linker to develop nanosponge-loaded hydrogel for local detoxification in bacterial infection [145]. Thus, functionalization of the nanosponge will help to potentiate membrane-toxin affinity but needs to be explored for systemic detoxification in experimental sepsis models.

### Future prospectives and conclusions

Sepsis is a condition involving complex pathophysiology with a significant risk of life-threatening organ dysfunction [1, 2, 51]. A more in-depth understanding of sepsis has led to an updated definition and is considered as a global health priority [6, 51]. Notably, a major clinical challenge in sepsis management is the increasing resistance to antibiotics, which, in some cases, leaves patients with no therapeutic options [146]. Nanotechnology-based approaches are currently viewed as an attractive therapeutic strategy that may be implemented to overcome challenges associated with sepsis management, due to its inherent ability to conquer bacterial resistance, and pharmacokinetic optimization as well. Data and reports presented in this review provide recent advancements and perspectives in sepsis diagnosis and therapeutics using nanoparticles or targeted drug delivery using nanotechnology. This report also provides an overview of feasibility, uniqueness and comparative analysis of diagnostic approaches, mainstream therapy such as antibiotics and emerging adjuvant therapies such as anti-inflammatory, antioxidant, antitoxin and blood detoxification therapies.

Early diagnosis and thus, early initiation of appropriate treatment is most critical in improving outcomes sepsis. However, due to the lack of sepsis specific signs and symptoms, sepsis diagnosis is often based on clinical experiences and patient history. The reported studies demonstrated that nanoparticle-based biosensors provide a rapid, specific and sensitive means of diagnosis of a broad range of biomarkers and infectious pathogen detection. Further, a recent study by Lee et al. (2020) showed that dual methods of nanosensors could be combined for ROS detection [84]; however, it remains indefinable for combination for different nanosensor mechanisms in the detection of multiple biomarkers. Reliance on a single biomarker for sepsis is challenging as commonly used biomarkers such as CRP and PCT display altered expression in other diseases such as trauma, surgery, and heatstroke [15]. It is noteworthy that efforts have been made with regards to the detection of combinations of biomarkers in clinical studies, which display greater diagnostic accuracy in differentiating bacterial versus nonbacterial associated inflammation [147], organ dysfunction, shock, and in-hospital mortality [148]. These reports provided further motivation for the development of a biomarker panel using nanoparticles to provide improved detection times coupled with accuracy. Apart from this, several emerging biomarkers are being validated for sepsis, including CitH3, soluble form of triggering receptor expressed on myeloid cells-1 (TREM-1), soluble urokinase-type plasminogen, receptor (suPAR), pro-adrenomedullin (pro-ADM), and presepsin, which have shown some prognostic and diagnostic value [15]. Based on our appraisal of findings from reported data, we expected that nanotechnology-based diagnosis of these emerging biomarkers could be achieved at even lower limits of detections.

Speed and sensitivity of nano diagnostics facilitate and guides the early initiation of antibiotic therapy clinically. Antibacterial sensitivity which is a major determinant of antibiotic choice remains unaddressed, with increasing reliance on time-consuming conventional methods. Sepsis involves hemodynamic alterations in patients that imposes a challenge in attaining desired antibacterial pharmacokinetic targets. This, in turn, leads to suboptimal dosing, treatment failure and development of resistance. Further, infections with MDR pathogens or polymicrobial infections which are more frequently found in a sepsis patient necessitates the implementation of combination therapies, based on susceptibility. Data on such nano-antibiotic combination therapies is limited, and more studies are needed to explore such combinations in nano-formulations. Many nano-formulations have been reported in which surface functionalization with antimicrobial peptides showed targeted delivery with potent antimicrobial activities. Such antimicrobial peptide containing nano-formulations can deliver antibiotics at the desired site (bacterial cell wall/ membranes, cytoplasm or nucleus) and potentiate bacterial killing, but these results need validation in well-designed clinical studies.

Importantly, the knowledge that sepsis involves dysregulated host immune and inflammatory responses; has increased research the evaluation of nano-formulations of adjuvant therapies such as anti-inflammatory, antioxidant, antitoxin and blood purification therapies. Amongst all therapies, notably, nano-formulations of anti-inflammatory agents were evaluated in multiple preclinical and clinical studies. However, due to the involvement of multiple signalling pathways and crosstalk, agents targeting specific pro-inflammatory cytokines, will not be able to achieve expected outcomes and, thus approaches targeting multiple pathways or biomolecules central to cytokine production will be warranted. In a recently published report by Wang et al. (2018), early sepsis diagnosis was combined with simultaneous extracorporeal blood disinfection, by using iron oxide magnetic nanoparticles functionalized with chlorin e6 molecules, and bacterial species-identifiable aptamers (Fe_3_O_4_-Ce6-Apt) [34]. The study demonstrated that the Fe_3_O_4_-Ce6-Apt nanosystem was sensitive (around 10 CFU/ml), rapid (within 1.5 h) and efficiently detected mono- or polymicrobial infection in a mouse model. Thus, a combination of diagnostic and extracorporeal blood disinfection with a newly developed nanosystem can be the beginning of a new era in sepsis diagnosis and management.

To conclude, sepsis is a condition characterised by uncontrolled immune and inflammatory responses and have several challenges in its diagnosis and management. Numerous nanoparticle-based diagnosis and therapeutics alternatives have been evaluated in various in vitro and in vivo sepsis models with promising results. Besides nano-formulations of antibiotic, adjuvant therapies and their targeted delivery with nano-formulations have also been evaluated and have some role in sepsis management. It would be not surprising if such therapies using nanotechnology applications became available for in-clinic use.

## Data Availability

Not applicable.

## References

[1] Singer M, Deutschman CS, Seymour CW, Shankar-Hari M, Annane D, Bauer M (2016). The third international consensus definitions for sepsis and septic shock (Sepsis-3). JAMA.

[2] Sartelli M, Kluger Y, Ansaloni L, Hardcastle TC, Rello J, Watkins RR (2018). Raising concerns about the Sepsis-3 definitions. World J Emerg Surg.

[3] WHA. Improving the prevention, diagnosis and clinical management of sepsis. Vol. 140, World Health Organization. https://www.who.int/servicedeliverysafety/areas/sepsis/en/. Accessed 27 Apr 2020.

[4] Rudd KE, Johnson SC, Agesa KM, Shackelford KA, Tsoi D, Kievlan DR (2020). Global, regional, and national sepsis incidence and mortality, 1990–2017: analysis for the Global Burden of Disease Study. Lancet.

[5] CDC. Sepsis. https://www.cdc.gov/sepsis/index.html. Accessed on 4 May 2020.

[6] World Health Organization (WHO). Sepsis 2018. https://www.who.int/news-room/fact-sheets/detail/sepsis. Accessed 14 Feb 2020.

[7] Schultz MJ, Dunser MW, Dondorp AM, Adhikari NKJ, Iyer S, Kwizera A (2017). Current challenges in the management of sepsis in ICUs in resource-poor settings and suggestions for the future. Intensive Care Med.

[8] Arentz M, Yim E, Klaff L, Lokhandwala S, Riedo FX, Chong M (2020). Characteristics and outcomes of 21 critically ill patients with COVID-19 in Washington State. JAMA.

[9] Bhatraju PK, Ghassemieh BJ, Nichols M, Kim R, Jerome KR, Nalla AK (2020). Covid-19 in critically ill patients in the Seattle Region—case series. N Engl J Med.

[10] Guo T, Fan Y, Chen M, Wu X, Zhang L, He T (2020). Cardiovascular implications of fatal outcomes of patients with coronavirus disease 2019 (COVID-19). JAMA Cardiol.

[11] Kumar S, Tripathy S, Jyoti A, Singh SG (2019). Recent advances in biosensors for diagnosis and detection of sepsis: a comprehensive review. Biosens Bioelectron.

[12] Limongi D, D’Agostini C, Ciotti M (2016). New sepsis biomarkers. Asian Pac J Trop Biomed.

[13] Ifedayo Kuye CR. Spotlight: Overdiagnosis and Delay: Challenges in Sepsis Diagnosis. https://psnet.ahrq.gov/web-mm/spotlight-overdiagnosis-and-delay-challenges-sepsis-diagnosis. Accessed 7 May 2020.

[14] Singer M (2019). Biomarkers for sepsis—past, present and future. Qatar Med J.

[15] Henriquez-Camacho C, Losa J (2014). Biomarkers for Sepsis. Biomed Res Int..

[16] Pierrakos C, Vincent J-L (2010). Sepsis biomarkers: a review. Crit Care.

[17] Leligdowicz A, Matthay MA (2019). Heterogeneity in sepsis: new biological evidence with clinical applications. Crit Care.

[18] Gyawali B, Ramakrishna K, Dhamoon AS (2019). Sepsis: the evolution in definition, pathophysiology, and management. SAGE Open Med.

[19] Gotts JE, Matthay MA (2016). Sepsis: pathophysiology and clinical management. BMJ.

[20] Hotchkiss RS, Moldawer LL, Opal SM, Reinhart K, Turnbull IR, Vincent J-L (2016). Sepsis and septic shock. Nat Rev Dis Prim.

[21] Cariou A, Vinsonneau C, Dhainaut J-F (2004). Adjunctive therapies in sepsis: an evidence-based review. Crit Care Med.

[22] Martin-Loeches I, Levy MM, Artigas A (2015). Management of severe sepsis: advances, challenges, and current status. Drug Des Devel Ther.

[23] Molnár Z, Giamarellos-Bourboulis EJ, Kumar A, Nierhaus A (2016). Sepsis: diagnostic and therapeutic challenges. Biomed Res Int.

[24] Alhazzani W, Møller MH, Arabi YM, Loeb M, Gong MN, Fan E, Sepsis S (2019). Surviving Sepsis Campaign: guidelines on the management of critically ill adults with coronavirus disease COVID-19. Crit Care Med.

[25] Capsoni N, Bellone P, Aliberti S, Sotgiu G, Pavanello D, Visintin B (2019). Prevalence, risk factors and outcomes of patients coming from the community with sepsis due to multidrug resistant bacteria. Multidiscip Respir Med.

[26] Laxminarayan R, Matsoso P, Pant S, Brower C, Røttingen J-A, Klugman K (2016). Access to effective antimicrobials: a worldwide challenge. Lancet.

[27] Li G, Bielicki JA, Ahmed ASMNU, Islam MS, Berezin EN, Gallacci CB (2020). Towards understanding global patterns of antimicrobial use and resistance in neonatal sepsis: insights from the NeoAMR network. Arch Dis Child..

[28] Uppu DSSM, Ghosh C, Haldar J (2015). Surviving sepsis in the era of antibiotic resistance: are there any alternative approaches to antibiotic therapy?. Microb Pathog.

[29] Charlton M, Thompson JP (2019). Pharmacokinetics in sepsis. BJA Educ.

[30] Selvaraj V, Manne ND, Arvapalli R, Rice KM, Nandyala G, Fankenhanel E (2015). Effect of cerium oxide nanoparticles on sepsis induced mortality and NF-κB signaling in cultured macrophages. Nanomedicine.

[31] Claxton A, Papafilippou L, Hadjidemetriou M, Kostarelos K, Dark P (2020). The challenge of recognising sepsis: future nanotechnology solutions. J Intensive Care Soc.

[32] Zhu X, Radovic-Moreno AF, Wu J, Langer R, Shi J (2014). Nanomedicine in the management of microbial infection—overview and perspectives. Nano Today.

[33] Li H, Yang T, Zhou H, Du J, Zhu B, Sun Z (2017). Emodin combined with nanosilver inhibited sepsis by anti-inflammatory protection. Front Pharmacol.

[34] Wang J, Wu H, Yang Y, Yan R, Zhao Y, Wang Y (2018). Bacterial species-identifiable magnetic nanosystems for early sepsis diagnosis and extracorporeal photodynamic blood disinfection. Nanoscale.

[35] Gao W, Xiong Y, Li Q, Yang H (2017). Inhibition of toll-like receptor signaling as a promising therapy for inflammatory diseases: a journey from molecular to nano therapeutics. Front Physiol.

[36] Khan ST, Musarrat J, Al-Khedhairy AA (2016). Countering drug resistance, infectious diseases, and sepsis using metal and metal oxides nanoparticles: current status. Colloids Surfaces B Biointerfaces.

[37] Yuk SA, Sanchez-Rodriguez DA, Tsifansky MD, Yeo Y (2018). Recent advances in nanomedicine for sepsis treatment. Ther Deliv.

[38] Cao C, Yu M, Chai Y (2019). Pathological alteration and therapeutic implications of sepsis-induced immune cell apoptosis. Cell Death Dis.

[39] Cavaillon J-M, Singer M, Skirecki T (2020). Sepsis therapies: learning from 30 years of failure of translational research to propose new leads. EMBO Mol Med.

[40] Teggert A, Datta H, Ali Z (2020). Biomarkers for point-of-care diagnosis of sepsis. Micromachines (Basel).

[41] Choi H, Kim Y, Mirzaaghasi A, Heo J, Kim YN, Shin JH (2020). Exosome-based delivery of super-repressor IκBα relieves sepsis-associated organ damage and mortality. Sci Adv..

[42] Hotchkiss RS, Monneret G, Payen D (2013). Sepsis-induced immunosuppression: from cellular dysfunctions to immunotherapy. Nat Rev Immunol.

[43] Needham DM, Davidson J, Cohen H, Hopkins RO, Weinert C, Wunsch H (2012). Improving long-term outcomes after discharge from intensive care unit: report from a stakeholders’ conference. Crit Care Med.

[44] Delano MJ, Ward PA (2016). Sepsis-induced immune dysfunction: can immune therapies reduce mortality?. J Clin Invest.

[45] Evans T (2018). Diagnosis and management of sepsis. Clin Med.

[46] Deutschman CS, Tracey KJ (2014). Sepsis: current dogma and new perspectives. Immunity.

[47] Vincent J-L (2016). The clinical challenge of sepsis identification and monitoring. PLoS Med.

[48] Carrigan SD, Scott G, Tabrizian M (2004). Toward resolving the challenges of sepsis diagnosis. Clin Chem.

[49] Gunsolus IL, Sweeney TE, Liesenfeld O, Ledeboer NA (2019). Diagnosing and managing sepsis by probing the host response to infection: advances, opportunities, and challenges. J Clin Microbiol..

[50] Lazăr A, Georgescu AM, Vitin A, Azamfirei L (2019). Precision medicine and its role in the treatment of sepsis: a personalised view. J Crit Care Med.

[51] Rhodes A, Evans LE, Alhazzani W, Levy MM, Antonelli M, Ferrer R (2017). Surviving sepsis campaign: international guidelines for management of sepsis and septic shock: 2016. Intensive Care Med.

[52] Marik PE, Farkas JD (2018). The changing paradigm of sepsis: early diagnosis, early antibiotics, early pressors, and early adjuvant treatment. Crit Care Med.

[53] De Backer D, Cecconi M, Lipman J, Machado F, Myatra SN, Ostermann M (2019). Challenges in the management of septic shock: a narrative review. Intensive Care Med.

[54] Garnacho-Montero J, Garcia-Garmendia JL, Barrero-Almodovar A, Jimenez-Jimenez FJ, Perez-Paredes C, Ortiz-Leyba C (2003). Impact of adequate empirical antibiotic therapy on the outcome of patients admitted to the intensive care unit with sepsis*. Crit Care Med.

[55] Busani S, Roat E, Serafini G, Mantovani E, Biagioni E, Girardis M (2017). The role of adjunctive therapies in septic shock by gram negative MDR/XDR infections. Can J Infect Dis Med Microbiol..

[56] Buxton DB (2009). Nanomedicine for the management of lung and blood diseases. Nanomedicine.

[57] Li YCE, Lee IC (2020). The current trends of biosensors in tissue engineering. Biosensors.

[58] Bhalla N, Jolly P, Formisano N, Estrela P (2016). Introduction to biosensors. Essays Biochem.

[59] Samraj RS, Zingarelli B, Wong HR (2013). Role of biomarkers in sepsis care. Shock.

[60] van Engelen TSR, Wiersinga WJ, Scicluna BP, van der Poll T (2018). Biomarkers in sepsis. Crit Care Clin.

[61] Faridbod F, Gupta VK, Zamani HA (2011). Electrochemical Sensors and Biosensors. Int J Electrochem.

[62] Pepys MB, Hirschfield GM (2003). C-reactive protein: a critical update. J Clin Invest.

[63] Póvoa P, Coelho L, Almeida E, Fernandes A, Mealha R, Moreira P (2005). C-reactive protein as a marker of infection in critically ill patients. Clin Microbiol Infect.

[64] Ibupoto ZH, Jamal N, Khun K, Willander M (2012). Development of a disposable potentiometric antibody immobilized ZnO nanotubes based sensor for the detection of C-reactive protein. Sens Actuators B Chem.

[65] Shukla P, Dwivedi P, Gupta PK, Mishra PR (2014). Optimization of novel tocopheryl acetate nanoemulsions for parenteral delivery of curcumin for therapeutic intervention of sepsis. Expert Opin Drug Deliv.

[66] Liu A, Wang X (2015). Amperometric immunosensor of procalcitonin based on amplification strategy of ferrocene-modified gold nanoparticles. Int J Electrochem Sci.

[67] Liu P, Li C, Zhang R, Tang Q, Wei J, Lu Y (2019). An ultrasensitive electrochemical immunosensor for procalcitonin detection based on the gold nanoparticles-enhanced tyramide signal amplification strategy. Biosens Bioelectron.

[68] Sánchez-Tirado E, Salvo C, González-Cortés A, Yáñez-Sedeño P, Langa F, Pingarrón JM (2017). Electrochemical immunosensor for simultaneous determination of interleukin-1 beta and tumor necrosis factor alpha in serum and saliva using dual screen printed electrodes modified with functionalized double-walled carbon nanotubes. Anal Chim Acta.

[69] Mahmudunnabi RG, Farhana FZ, Kashaninejad N, Firoz SH, Shim Y-B, Shiddiky MJA (2020). Nanozyme-based electrochemical biosensors for disease biomarker detection. Analyst.

[70] Xie J, Tang M-Q, Chen J, Zhu Y-H, Lei C-B, He H-W (2020). A sandwich ELISA-like detection of C-reactive protein in blood by citicoline-bovine serum albumin conjugate and aptamer-functionalized gold nanoparticles nanozyme. Talanta.

[71] Das R, Dhiman A, Kapil A, Bansal V, Sharma TK (2019). Aptamer-mediated colorimetric and electrochemical detection of *Pseudomonas aeruginosa* utilizing peroxidase-mimic activity of gold NanoZyme. Anal Bioanal Chem.

[72] Li H, Sun Y, Elseviers J, Muyldermans S, Liu S, Wan Y (2014). A nanobody-based electrochemiluminescent immunosensor for sensitive detection of human procalcitonin. Analyst.

[73] Liu J, Quan L, Yu X, Wang L (2019). Quantitative detection of procalcitonin using an electrochemical immunosensor based on MoO3/Au@rGO nanocomposites. Analyst.

[74] Wang J, Liu G, Engelhard MH, Lin Y (2006). Sensitive immunoassay of a biomarker tumor necrosis factor-α based on poly(guanine)-functionalized silica nanoparticle label. Anal Chem..

[75] Yin Z, Liu Y, Jiang LP, Zhu JJ (2011). Electrochemical immunosensor of tumor necrosis factor α based on alkaline phosphatase functionalized nanospheres. Biosens Bioelectron.

[76] Molinero-Fernández Á, Arruza L, López MÁ, Escarpa A (2020). On-the-fly rapid immunoassay for neonatal sepsis diagnosis: C-reactive protein accurate determination using magnetic graphene-based micromotors. Biosens Bioelectron.

[77] Mocan T, Matea CT, Pop T, Mosteanu O, Buzoianu AD, Puia C (2017). Development of nanoparticle-based optical sensors for pathogenic bacterial detection. J Nanobiotechnol.

[78] Mylonakis E, Clancy CJ, Ostrosky-Zeichner L, Garey KW, Alangaden GJ, Vazquez JA (2015). T2 magnetic resonance assay for the rapid diagnosis of candidemia in whole blood: a clinical trial. Clin Infect Dis.

[79] Neely LA, Audeh M, Phung NA, Min M, Suchocki A, Plourde D (2013). T2 magnetic resonance enables nanoparticle-mediated rapid detection of candidemia in whole blood. Sci Transl Med.

[80] Mylonakis E, Zacharioudakis IM, Clancy CJ, Hong Nguyen M, Pappas PG (2018). Efficacy of T2 magnetic resonance assay in monitoring candidemia after initiation of antifungal therapy: The serial therapeutic and antifungal monitoring protocol (STAMP) trial. J Clin Microbiol.

[81] Hu J, Zhang ZL, Wen CY, Tang M, Wu LL, Liu C (2016). Sensitive and quantitative detection of C-reaction protein based on immunofluorescent nanospheres coupled with lateral flow test strip. Anal Chem.

[82] Kitayama Y, Takeuchi T (2014). Localized surface plasmon resonance nanosensing of C-reactive protein with poly(2-methacryloyloxyethyl phosphorylcholine)-grafted gold nanoparticles prepared by surface-initiated atom transfer radical polymerization. Anal Chem.

[83] Wong R, Shou J, Wang Y. Probing sepsis and sepsis-like conditions using untargeted SPIO nanoparticles. Annu Int Conf IEEE Eng Med Biol Soc. 2010;2010:3053–6. 10.1109/IEMBS.2010.5626123. 10.1109/IEMBS.2010.5626123 21095733

[84] Lee DY, Kang S, Lee Y, Kim JY, Yoo D, Jung W (2020). PEGylated bilirubin-coated iron oxide nanoparticles as a biosensor for magnetic relaxation switching-based ros detection in whole blood. Theranostics.

[85] Deng Q, Pan B, Alam HB, Liang Y, Wu Z, Liu B (2020). Citrullinated histone H3 as a therapeutic target for endotoxic shock in mice. Front Immunol.

[86] Pan B, Alam HB, Chong W, Mobley J, Liu B, Deng Q (2017). CitH3: a reliable blood biomarker for diagnosis and treatment of endotoxic shock. Sci Rep.

[87] Park Y, Ryu B, Deng Q, Pan B, Song Y, Tian Y (2020). An integrated plasmo-photoelectronic nanostructure biosensor detects an infection biomarker accompanying cell death in neutrophils. Small.

[88] Kankala RK, Lin WZ, Lee CH (2020). Combating antibiotic resistance through the synergistic effects of mesoporous silica-based hierarchical nanocomposites. Nanomaterials.

[89] Hussain S, Joo J, Kang J, Kim B, Braun GB, She Z-G (2018). Antibiotic-loaded nanoparticles targeted to the site of infection enhance antibacterial efficacy. Nat Biomed Eng.

[90] Shaker MA, Shaaban MI (2017). Formulation of carbapenems loaded gold nanoparticles to combat multi-antibiotic bacterial resistance: In vitro antibacterial study. Int J Pharm.

[91] Abdelkader A, El-Mokhtar MA, Abdelkader O, Hamad MA, Elsabahy M, El-Gazayerly ON (2017). Ultrahigh antibacterial efficacy of meropenem-loaded chitosan nanoparticles in a septic animal model. Carbohydr Polym.

[92] Yadav V, Talwar P (2019). Repositioning of fluoroquinolones from antibiotic to anti-cancer agents: an underestimated truth. Biomed Pharmacother.

[93] Dalhoff A, Shalit I (2003). Immunomodulatory effects of quinolones. Lancet Infect Dis.

[94] Zhang CY, Gao J, Wang Z (2018). Bioresponsive nanoparticles targeted to infectious microenvironments for sepsis management. Adv Mater.

[95] Yang Y, Ding Y, Fan B, Wang Y, Mao Z, Wang W (2020). Inflammation-targeting polymeric nanoparticles deliver sparfloxacin and tacrolimus for combating acute lung sepsis. J Control Release.

[96] Mishra PR (2011). An investigation on the approach to target lipopolysaccharide through polymeric capped nano-structured formulation for the management of sepsis. J Biomed Nanotechnol.

[97] Jain V, Shukla P, Pal R, Mishra PR (2014). Cationic nanoemulsions bearing ciprofloxacin surf-plexes enhances its therapeutic efficacy in conditions of *E. coli* induced peritonitis and sepsis. Pharm Res..

[98] Handa M, Sharma A, Verma RK, Shukla R (2019). Polycaprolactone based nano-carrier for co-administration of moxifloxacin and rutin and its In-vitro evaluation for sepsis. J Drug Deliv Sci Technol.

[99] Hollmann A, Martinez M, Maturana P, Semorile LC, Maffia PC (2018). Antimicrobial peptides: interaction with model and biological membranes and synergism with chemical antibiotics. Front Chem.

[100] Lam SJ, O’Brien-Simpson NM, Pantarat N, Sulistio A, Wong EHH, Chen YY (2016). Combating multidrug-resistant Gram-negative bacteria with structurally nanoengineered antimicrobial peptide polymers. Nat Microbiol.

[101] Fan X, Fan J, Wang X, Wu P, Wu G (2015). S-thanatin functionalized liposome potentially targeting on Klebsiella pneumoniae and its application in sepsis mouse model. Front Pharmacol.

[102] Arunmanee W, Pathania M, Solovyova AS, Le Brun AP, Ridley H, Baslé A (2016). Gram-negative trimeric porins have specific LPS binding sites that are essential for porin biogenesis. Proc Natl Acad Sci U S A.

[103] Biswaro LS, de Sousa MGC, Rezende TMB, Dias SC, Franco OL (2018). Antimicrobial peptides and nanotechnology, recent advances and challenges. Front Microbiol..

[104] Hotchkiss RS, Monneret G, Payen D (2013). Immunosuppression in sepsis: a novel understanding of the disorder and a new therapeutic approach. Lancet Infect Dis.

[105] Meisel C, Schefold JC, Pschowski R, Baumann T, Hetzger K, Gregor J (2009). Granulocyte-macrophage colony-stimulating factor to reverse sepsis-associated immunosuppression. Am J Respir Crit Care Med.

[106] Presneill JJ, Harris T, Stewart AG, Cade JF, Wilson JW (2002). A randomized phase ii trial of granulocyte-macrophage colony-stimulating factor therapy in severe sepsis with respiratory dysfunction. Am J Respir Crit Care Med.

[107] Hou X, Zhang X, Zhao W, Zeng C, Deng B, McComb DW (2020). Vitamin lipid nanoparticles enable adoptive macrophage transfer for the treatment of multidrug-resistant bacterial sepsis. Nat Nanotechnol.

[108] Swamy MK, Akhtar MS, Sinniah UR (2016). Antimicrobial properties of plant essential oils against human pathogens and their mode of action: an updated review. Evid Based Complement Alternat Med.

[109] Montagu A, Saulnier P, Cassissa V, Rossines E, Eveillard M, Joly-Guillou ML (2014). Aromatic and terpenic compounds loaded in lipidic nanocapsules: activity against multi-drug resistant acinetobacter baumannii assessed in vitro and in a murine model of sepsis. J Nanomed Nanotechnol.

[110] Kuthati Y, Kankala RK, Lin SX, Weng CF, Lee CH (2015). pH-triggered controllable release of silver-indole-3 acetic acid complexes from mesoporous silica nanoparticles (IBN-4) for effectively killing malignant bacteria. Mol Pharm.

[111] Liu M, Fang F, Song X, Yu F, Li F, Shi X (2016). The first visually observable three-mode antibiotic switch and its relative 3D printing assisted applications. J Mater Chem B.

[112] Liu MZ, Zhang HQ, Song XW, Wei CC, Xiong ZF, Yu F (2018). NaCl: for the safer in vivo use of antibacterial silver based nanoparticles. Int J Nanomed.

[113] Kuthati Y, Kankala RK, Busa P, Lin SX, Deng JP, Mou CY (2017). Phototherapeutic spectrum expansion through synergistic effect of mesoporous silica trio-nanohybrids against antibiotic-resistant gram-negative bacterium. J Photochem Photobiol B Biol.

[114] Amato E, Diaz-Fernandez YA, Taglietti A, Pallavicini P, Pasotti L, Cucca L (2011). Synthesis, characterization and antibacterial activity against gram positive and gram negative bacteria of biomimetically coated silver nanoparticles. Langmuir.

[115] Khanal M, Raks V, Issa R, Chernyshenko V, Barras A, Garcia Fernandez JM (2015). Selective antimicrobial and antibiofilm disrupting properties of functionalized diamond nanoparticles against *Escherichia coli* and *Staphylococcus aureus*. Part Part Syst Charact.

[116] Ding R, Meng Y, Ma X. The Central Role of the Inflammatory Response in Understanding the Heterogeneity of Sepsis-3. Tsirigotis P, editor. Biomed Res Int. 2018;2018:5086516. 10.1155/2018/5086516 PMC6011097 29977913

[117] Nedeva C, Menassa J, Puthalakath H (2019). Sepsis: inflammation is a necessary evil. Front Cell Dev Biol.

[118] Schulte W, Bernhagen J, Bucala R (2013). Cytokines in sepsis: potent immunoregulators and potential therapeutic targets—an updated view. Mediators Inflamm..

[119] Rodriguez Lavado J, Sestito SE, Cighetti R, Aguilar Moncayo EM, Oblak A, Lainšček D (2014). Trehalose- and glucose-derived glycoamphiphiles: small-molecule and nanoparticle toll-like receptor 4 (TLR4) modulators. J Med Chem.

[120] Yang H, Fung SY, Xu S, Sutherland DP, Kollmann TR, Liu M (2015). Amino acid-dependent attenuation of toll-like receptor signaling by peptide-gold nanoparticle hybrids. ACS Nano.

[121] Taratummarat S, Sangphech N, Vu CTB, Palaga T, Ondee T, Surawut S (2018). Gold nanoparticles attenuates bacterial sepsis in cecal ligation and puncture mouse model through the induction of M2 macrophage polarization. BMC Microbiol.

[122] Yang H, Kozicky L, Saferali A, Fung SY, Afacan N, Cai B (2016). Endosomal pH modulation by peptide-gold nanoparticle hybrids enables potent anti-inflammatory activity in phagocytic immune cells. Biomaterials.

[123] Fratoddi I, Venditti I, Cametti C, Russo MV (2015). The puzzle of toxicity of gold nanoparticles. The case-study of HeLa cells. Toxicol Res..

[124] Foit L, Thaxton CS (2016). Synthetic high-density lipoprotein-like nanoparticles potently inhibit cell signaling and production of inflammatory mediators induced by lipopolysaccharide binding Toll-like receptor 4. Biomaterials.

[125] Xu Y, Li Y, Liu X, Pan Y, Sun Z, Xue Y (2019). SPIONs enhances IL-10-producing macrophages to relieve sepsis via Cav1-Notch1/HES1-mediated autophagy. Int J Nanomed.

[126] Spence S, Greene MK, Fay F, Hams E, Saunders SP, Hamid U (2015). Targeting Siglecs with a sialic acid-decorated nanoparticle abrogates inflammation. Sci Transl Med.

[127] Xu X, Rui S, Chen C, Zhang G, Li Z, Wang J (2020). Protective effects of astragalus polysaccharide nanoparticles on septic cardiac dysfunction through inhibition of TLR4/NF-κB signaling pathway. Int J Biol Macromol.

[128] Chen G, Xu Y (2018). Biosynthesis of cerium oxide nanoparticles and their effect on lipopolysaccharide (LPS) induced sepsis mortality and associated hepatic dysfunction in male Sprague Dawley rats. Mater Sci Eng C.

[129] Wang J, Wang H, Zhu R, Liu Q, Fei J, Wang S (2015). Anti-inflammatory activity of curcumin-loaded solid lipid nanoparticles in IL-1β transgenic mice subjected to the lipopolysaccharide-induced sepsis. Biomaterials.

[130] Chatterjee S. Oxidative stress, inflammation, and disease. In: Dziubla, T Butterfield DA, editors. Oxidative stress and biomaterials. Academic Press, Cambridge;2016. pp 35–58.

[131] Prauchner CA (2017). Oxidative stress in sepsis: pathophysiological implications justifying antioxidant co-therapy. Burns.

[132] Nagar H, Piao S, Kim CS (2018). Role of mitochondrial oxidative stress in sepsis. Acute Crit Care.

[133] Naik E, Dixit VM (2011). Mitochondrial reactive oxygen species drive proinflammatory cytokine production. J Exp Med.

[134] Li Volti G, Musumeci T, Pignatello R, Murabito P, Barbagallo I, Carbone C (2012). Antioxidant potential of different melatonin-loaded nanomedicines in an experimental model of sepsis. Exp Biol Med.

[135] Wang D, Yin Y, Yao Y (2014). Advances in sepsis-associated liver dysfunction. Burn Trauma.

[136] Chen G, Deng H, Song X, Lu M, Zhao L, Xia S (2017). Reactive oxygen species-responsive polymeric nanoparticles for alleviating sepsis-induced acute liver injury in mice. Biomaterials.

[137] Kang JH, Um E, Diaz A, Driscoll H, Rodas MJ, Domansky K (2015). Optimization of pathogen capture in flowing fluids with magnetic nanoparticles. Small.

[138] Herrmann IK, Urner M, Koehler FM, Hasler M, Roth-Z’graggen B, Grass RN (2010). Blood purification using functionalized core/shell nanomagnets. Small..

[139] Lee JJ, Jeong KJ, Hashimoto M, Kwon AH, Rwei A, Shankarappa SA (2014). Synthetic ligand-coated magnetic nanoparticles for microfluidic bacterial separation from blood. Nano Lett.

[140] Kang JH, Super M, Yung CW, Cooper RM, Domansky K, Graveline AR (2014). An extracorporeal blood-cleansing device for sepsis therapy. Nat Med.

[141] Fang RH, Luk BT, Hu CMJ, Zhang L (2015). Engineered nanoparticles mimicking cell membranes for toxin neutralization. Adv Drug Deliv Rev.

[142] Donnell ML, Lyon AJ, Mormile MR, Barua S (2016). Endotoxin hitchhiking on polymer nanoparticles. Nanotechnology.

[143] Hu CMJ, Fang RH, Copp J, Luk BT, Zhang L (2013). A biomimetic nanosponge that absorbs pore-forming toxins. Nat Nanotechnol.

[144] Thamphiwatana S, Angsantikul P, Escajadillo T, Zhang Q, Olson J, Luk BT (2017). Macrophage-like nanoparticles concurrently absorbing endotoxins and proinflammatory cytokines for sepsis management. Proc Natl Acad Sci USA.

[145] Wang F, Gao W, Thamphiwatana S, Luk BT, Angsantikul P, Zhang Q (2015). Hydrogel retaining toxin-absorbing nanosponges for local treatment of methicillin-resistant *Staphylococcus aureus* infection. Adv Mater.

[146] Friedman ND, Temkin E, Carmeli Y (2016). The negative impact of antibiotic resistance. Clin Microbiol Infect.

[147] Kofoed K, Andersen O, Kronborg G, Tvede M, Petersen J, Eugen-Olsen J (2007). Use of plasma C-reactive protein, procalcitonin, neutrophils, macrophage migration inhibitory factor, soluble urokinase-type plasminogen activator receptor, and soluble triggering receptor expressed on myeloid cells-1 in combination to diagnose infections. Crit Care.

[148] Shapiro NI, Trzeciak S, Hollander JE, Birkhahn R, Otero R, Osborn TM (2009). A prospective, multicenter derivation of a biomarker panel to assess risk of organ dysfunction, shock, and death in emergency department patients with suspected sepsis. Crit Care Med.

